# Circulating microRNA signatures as potential biomarkers differentiating diabetic, prediabetic, and healthy individuals

**DOI:** 10.3389/fendo.2025.1699100

**Published:** 2025-11-04

**Authors:** Hanifa J. Abu-Toamih Atamni, Guy Shapira, Rona Ortenberg, Maya Sultan, Orit Twito, Liat Yoseph-Barzilay, Noam Shomron, Gloria Rashid

**Affiliations:** 1 Clinical Research Laboratory, Meir Medical Center, Kefar Saba, Israel; 2 Immunology Laboratory, Meir Medical Center, Kefar Saba, Israel; 3 Department of Cell and Developmental Biology, Gray Faculty of Medical and Health Sciences, Tel Aviv University, Tel Aviv, Israel; 4 Edmond J. Safra Center for Bioinformatics, Tel Aviv University, Tel Aviv, Israel; 5 Hematology Laboratory, Meir Medical Center, Kefar Saba, Israel; 6 Endocrinology, Diabetes, and Metabolism Institute, Meir Medical Center, Kefar Saba, Israel

**Keywords:** type 2 diabetes, prediabetes, circulating microRNAs, biomarkers, glycemic control, vitamin d, platelet aggregation, sex differences

## Abstract

**Introduction:**

Circulating microRNAs (miRNAs) are small non-coding RNAs that regulate gene expression and play key roles in metabolic diseases, including diabetes. This study aimed to identify circulating miRNA signatures linked to glycemic status, vitamin D deficiency, aspirin consumption, and platelet activity in individuals with type 2 diabetes, prediabetes, and healthy controls.

**Methods:**

Plasma samples from 24 participants (14 with diabetes, 2 prediabetic, and 8 non-diabetic controls) were analyzed using next-generation sequencing to assess differences in miRNA expression.

**Results:**

Although principal component analysis showed no group separation, pairwise testing identified 131 miRNAs that were significantly altered between the diabetic and control groups, as well as 141 miRNAs between the prediabetic and control groups (FDR <0.05), with 56 overlapping between contrasts. Fourteen miRNAs in the pre-diabetic group and twelve in the diabetic group overlapped with literature-supported candidates, reinforcing their clinical relevance. In the direct diabetes vs. prediabetes comparison, no miRNAs passed FDR. Still, consistent trend-level decreases in diabetes were observed for hsa-miR-4776-5p, hsa-miR-6778-3p, and hsa-miR-5002-3p, which were among the most upregulated in prediabetes vs. controls. Sex-stratified analyses revealed distinct miRNA expression patterns, emphasizing the influence of biological sex on miRNA regulation in glucose metabolism. Correlation analyses highlighted miRNAs associated with glucose/HbA1c, alongside relationships among vitamin D status, glycemia, and platelet function.

**Discussion:**

Collectively, these data highlight the exploratory potential of circulating miRNAs as candidate early biomarkers for glycemic dysregulation, providing a foundation for future validation and development of improved diagnostic and preventive strategies for type 2 diabetes.

## Introduction

Diabetes is one of the leading non-communicable diseases, ranking fourth, and affects over half a billion (537 million) adults worldwide. It is estimated that the number of adults will reach 783 million by 2045 (1–3). This chronic metabolic disease is characterized by high blood sugar levels caused by insulin resistance or a lack of insulin, which can lead to serious health complications such as heart disease, stroke, and kidney failure (4). Over 90% of diabetes cases globally are Type 2 diabetes, which is rapidly becoming a major public health concern due to multiple polygenic and environmental factors like poor diet, sedentary lifestyle, increasing age, ethnicity, family history, and obesity (1). The diagnosis of Type 2 Diabetes Mellitus (T2DM) is based on fasting glucose levels, two-hour plasma glucose concentrations during an oral glucose tolerance test (OGTT), and/or measurements of hemoglobin A1c (HbA1c). Lower thresholds for these indicators are used to detect early stages of the disease. Individuals showing early signs of dysglycemia may have an increased risk of developing T2DM, requiring targeted preventive interventions. These early stages are often called pre-diabetes (PD) or intermediate hyperglycemia (IH) (5, 6). However, for clarity, the term pre-diabetes will be used throughout this manuscript.

Identifying biomarkers for predicting the onset and progression of diabetes is crucial for enabling early diagnosis and intervention. MicroRNAs (miRNAs), which are small non-coding RNAs approximately 22 nucleotides long, play a crucial role in post-transcriptional gene regulation and can serve as potential biomarkers for early detection due to their upstream position in regulatory cascades (7–9). They work by binding to specific sequences on target messenger RNAs, leading to either mRNA degradation or translation inhibition. MiRNAs regulate essential genes and signaling pathways involved in cellular processes such as insulin secretion, insulin sensitivity, pancreatic beta-cell function, glucose metabolism/homeostasis, platelet reactivity, and inflammation, all of which become dysregulated in diabetes (8, 10–13). Examining miRNA expression patterns in diabetic, pre-diabetic, and non-diabetic patients can provide vital insights into the development of diabetes (14). Additionally, analyzing miRNAs in diabetic patients can provide a deeper understanding of the molecular mechanisms underlying diabetes and its complications, facilitating the prediction of their development and enabling early interventions to mitigate their impact on health. Thanks to advances in molecular and computational methods, miRNAs offer advantages for early diagnosis, particularly because they can be detected at low levels (compared to proteins and metabolites) through amplification techniques such as qPCR and are more stable than mRNA (9, 15). Numerous studies have demonstrated altered expression of various microRNAs (miRNAs) in patients with type 2 diabetes (T2D) (16–19). A recent meta-analysis by Zhu et al. (2023) identified and validated 16 miRNAs (from 404 differentially expressed miRNAs across 156 studies) that are both statistically and biologically significant in relation to type 2 diabetes (20).

We included aspirin use in our sample selection because medications can influence circulating miRNA expression, especially those linked to cardiovascular and inflammatory pathways. Aspirin, commonly used for the prevention of cardiovascular disease, affects several microRNAs (miRNAs) related to vascular function, platelet activity, and inflammation, including hsa-miR-21, hsa-miR-126, and hsa-miR-155. According to Paseban et al. (2020), these miRNAs play a crucial role in the pathophysiology of cardiometabolic conditions (21). By identifying whether individuals were taking aspirin or not, we can control for this potential confounder and thus increase the accuracy of interpreting miRNA changes associated with diabetes.

Therefore, our study aims to explore variations in miRNA expression in blood plasma samples from individuals with diabetes compared to non-diabetic controls, building on the foundational research established by Sultan et al. (2019) in our laboratory. Sultan et al., 2019 investigated the relationship between platelet aggregation, vitamin D levels, and glycemic control in diabetic and pre-diabetic patients compared to healthy individuals, concluding that glycemic control is inversely related to high platelet aggregation and low vitamin D levels (22). Notably, the plasma samples analyzed here were originally collected in the study by Sultan et al. (2019), which focused on platelet aggregation, vitamin D, and glycemic control but did not include microRNA analysis. The novelty of the present study lies in applying next-generation sequencing to this archived cohort to profile circulating miRNAs, thereby expanding the scientific scope of the original dataset. In this study, we applied NGS to this archived cohort, we aimed to determine whether significant differences exist in miRNA expression between these groups, ultimately identifying new miRNAs that are directly related to T2DM (glucose and HbA1C levels) or T2DM complications (vitamin D deficiency and platelet activation). In the future, the study will validate these miRNAs in a larger sample and investigate their potential target genes and the associated biological processes.

## Materials and methods

### Ethical statement

This study was conducted in accordance with the principles of the Declaration of Helsinki and received approval from the institutional Helsinki Committee for Health Research Ethics at Meir Medical Center (protocol 0138-16-MMC). All participants provided written informed consent.

### Study cohort

This study utilized plasma samples previously collected in our laboratory from Sultan et al. (2019) to investigate the relationship between platelet aggregation, vitamin D levels, and HbA1c in healthy individuals and those with type 2 diabetes mellitus (T2DM). A total of 24 plasma samples were analyzed, including 14 diabetic subjects (mean age 65 ± 6.3 years), 2 pre-diabetic individuals (mean age 66 ± 7.8 years), and 8 healthy controls (mean age 64 ± 12 years). Inclusion and exclusion criteria are detailed in Sultan et al. (2019) (22). While the samples were previously described, their microRNA content has not been studied. In this work, we applied next-generation sequencing and bioinformatics pipelines to characterize circulating miRNA expression.

Clinical data included age, sex, BMI, medication use (such as vitamin D, multivitamins, anticoagulants, antiplatelet agents, and antiaggregants), and major comorbidities. Additionally, all samples were evaluated for blood biochemistry profiles [total cholesterol, high-density lipoprotein (HDL), calcium (Ca, mg/dL)], complete blood count (total platelets, mean platelet volume), HbA1c (%), and serum 25-hydroxyvitamin D [25(OH)D] levels, measured using standard automated clinical laboratory methods (22). These variables were included in the statistical assessment where possible, and subgroup analyses were performed by sex and aspirin exposure to partially control for confounding.

The summary of clinical and biochemical data for these plasma samples is provided in **Table 1**. This study aimed further to examine microRNA expression differences in diabetic versus healthy individuals using next-generation sequencing (NGS) and computational approaches, thereby building on the foundational work of Sultan et al. (2019).

**Table 1:** Clinical, biochemical, and demographic characteristics of the study cohort.

Characteristics	Total T2D patients (n=14)	Non-aspirin T2D patients (n=8)	Aspirin-associated T2D patients (n=6)	Pre-diabetic (n=2)	Healthy controls (n=8)
Male (n)	8	4	4	1	2
Female (n)	6	4	2	1	6
Age (years)	65.07	64.63 ± 2.55	65.67 ± 2.23	65.50 ± 7.78	63.50 ± 4.14
Diabetes Duration (years)	18.14 ± 2.36*	20.50 ± 3.43*	15.00 ± 2.91*	0.00 ± 0.00	0.00 ± 0.00
BMI (kg/m2)	30.30 ± 1.49	28.48 ± 1.25	32.73 ± 2.93	23.20 ± 2.55	25.80 ± 2.10
HbA1c (%)	7.59 ± 0.17*	7.84 ± 0.20*	7.26 ± 0.22*	5.90 ± 0.14	5.32 ± 0.09
FBG (mg/dL)	177.29 ± 8.46*	180.25 ± 12.13*	173.33 ± 12.40*	97.00 ± NA	86.86 ± 5.21
25(OH)D (nmol/L)	53.47 ± 4.70*	52.56 ± 6.25	54.68 ± 7.77	56.00 ± NA	78.16 ± 7.11
Ca (mg/dL)	9.29 ± 0.17	9.45 ± 0.25	9.09 ± 0.22	10.43 ± NA	9.05 ± 0.24
TG (mg/dL)	155.36 ± 23.37	137.50 ± 25.14	179.17 ± 43.97	218.00 ± NA	91.14 ± 19.89
Cr (mg/dL)	0.84 ± 0.05	0.89 ± 0.06	0.77 ± 0.07	0.90 ± NA	0.94 ± 0.14
HDL (mg/dL)	43.86 ± 2.88	43.49 ± 3.88	44.37 ± 4.71	46.30 ± NA	61.76 ± 7.19
Cholesterol (mg/dL)	151.57 ± 8.05	137.50 ± 9.43*	170.33 ± 10.17**	160.00 ± NA	180.14 ± 10.77
PLT (×10³/μL)	232.71 ± 11.72	218.38 ± 11.88	251.83 ± 21.03	220.50 ± 36.06	210.88 ± 14.22
Platelet Aggregation (%)	49.07 ± 9.28	73.26 ± 9.00*	16.82 ± 2.75**	89.60 ± NA	40.90 ± 12.07

This table summarizes the characteristics of 24 plasma samples analyzed in this study: 14 patients with type 2 diabetes mellitus (T2D), subdivided into non-aspirin users (n = 8) and aspirin users (n = 6), 2 pre-diabetic individuals, and 8 healthy controls. Parameters include sex, age, diabetes duration, body mass index (BMI), fasting blood glucose (FBG), glycated hemoglobin (HbA1c, %), serum 25-hydroxyvitamin D [25(OH)D], calcium (Ca, mg/dL), lipid profile [high-density lipoprotein (HDL), total cholesterol, triglycerides (TG)], creatinine (Cr, mg/dL), platelet count (PLT, ×10³/μL), and platelet aggregation (%). Data are presented as mean ± standard deviation (SD). *p < 0.05 vs. healthy controls; **p < 0.05 vs. non-aspirin T2D group. NA indicates data not available for the pre-diabetic group.

### MicroRNA extraction

Frozen plasma samples were incubated in a water bath at 37 °C until they were fully melted and the salts had dissolved. MicroRNA was extracted from plasma samples (initial volume of 200 µL) following the manufacturer’s instructions for the miRNAeasy Serum/Plasma Advanced Kit (Qiagen Catalog No. 217204) and quantified using a Nanodrop spectrophotometer (Nanodrop 2000, Thermo Scientific). MicroRNA concentrations ranged from 7.9 to 50.1 ng/µL and were stored at -80 °C until further analysis. All plasma samples were stored at −80 °C and subjected to a single thaw cycle prior to RNA extraction. Each RNA sample underwent quality-control assessment before library preparation and sequencing, and any that did not meet QC standards were excluded from analysis, including several pre-diabetic samples that failed QC (as noted in the Discussion). Long-term storage and freeze–thaw may nonetheless affect RNA integrity and are acknowledged as potential limitations.

### miRNA quantification and analysis

Raw sequencing data were processed using the nf-core/smrnaseq pipeline (version 2.4.0) (23). Briefly, the reads were trimmed and filtered with fastp (24) and then aligned with Bowtie to the miRBase reference. Downstream analysis was performed using R language libraries (version 4.4.2), with DESeq2 (version 1.48.1) (25) for differential expression and ggplot2 for the figures. All microRNA names were standardized according to miRBase version 22.1 nomenclature, using the species prefix “hsa-” to indicate *Homo sapiens*, and presented in the format hsa-miR-###-3p/5p throughout the manuscript, tables, and figures for consistency.

### Statistics

Statistical analysis employed paired t-tests, one-way ANOVA, and Pearson correlations to assess differences between groups, relationships among variables, and the significance of the results. Adjusted p-values (FDR) < 0.05 were considered statistically significant; results not meeting this threshold were classified as trend-level and interpreted as exploratory.

## Results

### Notable differences in miRNA expression among diabetic, pre-diabetic, and healthy individuals

**Figure 1:**
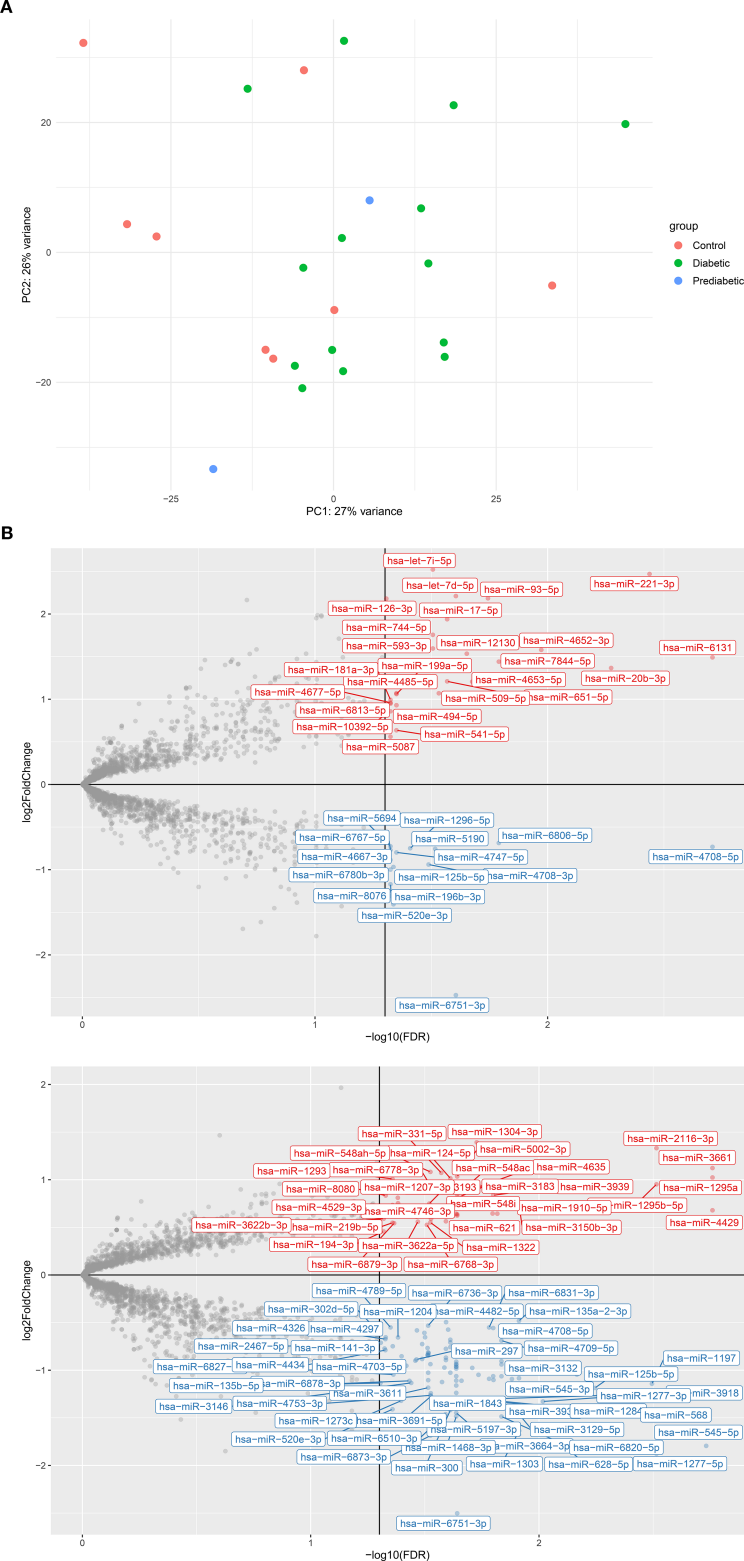
Analysis of varying hsa-miR expression in type 2 diabetes (T2D) and pre-diabetic patients compared to controls. **(A)** Principal component analysis (PCA) scatterplot of rlog-transformed hsa-miR expression profiles from 14 T2D patients (green), 2 pre-diabetic individuals (blue), and 8 controls (red), and 8 controls (green). **(B)** Volcano plots showing differentially expressed hsa-miRs between **(B.1)** T2D vs. controls **(B.2)** pre-diabetic vs. controls. Red points indicate significantly upregulated hsa-miRs, blue points indicate significantly downregulated hsa-miRs, and grey points represent non-significant changes. log_2_FC, log_2_ fold change; FDR, false discovery rate.

MicroRNA (miRNA) expression levels varied significantly (FDR < 0.05) among the diabetic, pre-diabetic, and healthy control groups (**Supplementary Table S1**). However, principal component analysis (PCA) of global expression profiles revealed no clear separation among the three groups (**Figure 1A**), indicating that factors other than disease status may influence overall miRNA variation.

Differential analysis between diabetic and healthy individuals identified 131 miRNAs with significantly altered expression (FDR < 0.05), of which 92 were downregulated and 39 were upregulated (**Figure 1B**.1). This broad downregulation pattern suggests suppression of miRNA-regulated pathways in diabetes. The most significantly downregulated miRNAs included hsa-miR-4708-5p (log_2_FC = –0.62, FDR = 9.4 × 10^-5^), hsa-miR-152-5p (log_2_FC = –0.80, FDR = 0.0012), hsa-miR-1197 (log_2_FC = –0.92, FDR = 0.0015), hsa-miR-4708-3p (log_2_FC = –0.84, FDR = 0.0038), and hsa-miR-125b-5p (log_2_FC = –0.92, FDR = 0.0044). Conversely, a subset of miRNAs showed significant upregulation, including hsa-miR-20b-3p (log_2_FC = 1.07, FDR = 0.0084), hsa-miR-4663 (log_2_FC = 0.74, FDR = 0.0125), hsa-miR-6079 (log_2_FC = 0.58, FDR = 0.0125), hsa-miR-516b-5p (log_2_FC = 0.47, FDR = 0.0125), and hsa-miR-541-5p (log_2_FC = 0.50, FDR = 0.0173).

Among the 131 differentially expressed miRNAs, 12 overlapped with the top 16 miRNAs reported in the recent meta-analysis by Zhu et al. (2023) (20), including hsa-miR-29a-3p, hsa-miR-221-3p, hsa-miR-126-3p, hsa-miR-26a-5p, hsa-miR-503-5p, hsa-miR-100-5p, hsa-miR-101-3p, hsa-miR-103a-3p, hsa-miR-122-5p, hsa-miR-199a-3p, hsa-miR-30b-5p, and hsa-miR-130a-3p (**Supplementary Table S2A**). **Supplementary Tables S2A, B** summarize the overlap between our findings and literature-validated miRNAs from Zhu et al. (2023), highlighting reproducibility across independent datasets. This overlap strengthens the clinical relevance of our findings and highlights their potential as circulating biomarkers for diabetes.

Similarly, we analyzed microRNA (miRNA) expression profiles in pre-diabetic individuals compared to healthy controls. Of the 2,647 miRNAs analyzed, 141 showed significant differential expression (FDR < 0.05), with 97 downregulated and 44 upregulated (**Figure 1B.2**). The most strongly downregulated miRNAs included hsa-miR-545-5p (log_2_FC = –1.79, FDR = 0.0019), hsa-miR-1277-5p (log_2_FC = –1.88, FDR = 0.0031), hsa-miR-1197 (log_2_FC = –1.14, FDR = 0.0032), hsa-miR-125b-5p (log_2_FC = –1.19, FDR = 0.0057), and hsa-miR-3132 (log_2_FC = –1.05, FDR = 0.0068). Conversely, significantly upregulated miRNAs included hsa-miR-4429 (log_2_FC = 0.68, FDR = 0.0017), hsa-miR-3661 (log_2_FC = 1.12, FDR = 0.0017), hsa-miR-1295a (log_2_FC = 1.02, FDR = 0.0017), hsa-miR-1295b-5p (log_2_FC = 0.95, FDR = 0.0031), and hsa-miR-2116-3p (log_2_FC = 1.33, FDR = 0.0031).

These results indicate that pre-diabetic individuals already exhibit a distinct miRNA expression signature relative to non-diabetic controls, suggesting early regulatory shifts associated with the development of diabetes. The complete list of significantly altered miRNAs is provided in **Supplementary Table S1B**. Among the 141 significantly dysregulated miRNAs, 14 overlapped with the top 16 literature-validated candidates highlighted by Zhu et al. (2023) (20) (**Supplementary Table S2B**). **Supplementary Table S2B** provides the corresponding overlap for the pre-diabetic versus control comparison. This overlap reinforces their exploratory relevance in early dysglycemia and supports their potential as candidate biomarkers pending validation for diabetes progression. Notably, 56 of the significantly dysregulated miRNAs overlapped between diabetic and pre-diabetic groups, underscoring a shared molecular signature across disease stages.

Next, differential expression analysis was performed to compare miRNA profiles between diabetic and pre-diabetic patients. No miRNAs met the threshold for statistical significance after adjusting for multiple testing (FDR < 0.05), emphasizing the similarity between these two groups. Several miRNAs displayed trend-level differences (uncorrected p < 0.001) that are considered exploratory findings rather than statistically significant results (**Supplementary Table S1C**). The most prominent were hsa-miR-4776-5p (log_2_FC = –0.65, FDR = 0.22), hsa-miR-6778-3p (log_2_FC = –1.02, FDR = 0.30), and hsa-miR-5002-3p (log_2_FC = –0.99, FDR = 0.32), all downregulated in diabetic compared to pre-diabetic individuals. Other miRNAs with suggestive differences included hsa-miR-1304-3p (log_2_FC = –1.23, FDR = 0.33) and hsa-miR-580-3p (log_2_FC = –0.88, FDR = 0.33). Interestingly, these same miRNAs were among the most upregulated in pre-diabetic individuals compared to healthy controls (**Supplementary Table S1B**), suggesting possible biological trends that require validation in larger cohorts.

### Functional enrichment of differentially expressed miRNAs

To explore the biological relevance of the 131 miRNAs that were differentially expressed between diabetic and non-diabetic patients, we conducted a functional enrichment analysis using the TAM 2.0 (Tool for Annotations of MicroRNAs; http://www.lirmed.com/tam2/)| (26, 27) platform. This analysis provided insights into the potential roles of these miRNAs in various physiological and pathological processes related to diabetes.

Several functional categories were significantly enriched among the differentially expressed miRNAs. Notably, glucose metabolism emerged as a relevant biological process (Fold enrichment = 2.62, FDR = 0.2287), involving hsa-miR-124-1, hsa-miR-124-2, hsa-miR-124-3, and both isoforms of hsa-miR-125b. Because this FDR value exceeds conventional significance thresholds, this pathway is considered a nominal enrichment and should be interpreted cautiously. These miRNAs have been previously associated with regulating insulin sensitivity, glucose homeostasis, and β-cell function (28).

The most significantly enriched category was embryonic development (Fold enrichment = 5.19, FDR = 0.0118), reflecting broader regulatory roles of these miRNAs in developmental gene networks. Other highly enriched categories included T-helper 17 (Th17) cell differentiation (Fold = 4.64, FDR = 0.0200), regulation of stem cells (Fold = 2.60, FDR = 0.0216), and inflammation (Fold = 2.23, FDR = 0.0314), all of which are relevant to the inflammatory and immune dysregulation observed in diabetes (29).

Furthermore, miRNAs were significantly associated with processes related to cell differentiation (Fold = 2.36, FDR = 0.1354), cell death (Fold = 2.07, FDR = 0.1631), and cell proliferation (Fold = 1.84, FDR = 0.3251). These enrichments are below statistical significance and are reported as exploratory findings that may indicate biological trends warranting further validation. These findings suggest a possible involvement of the identified miRNAs in the altered tissue regeneration and turnover observed in diabetes (30).

Other notable enriched categories included Osteogenesis (Fold = 2.74, FDR = 0.0335), which is consistent with bone fragility and impaired skeletal integrity in diabetic patients (31); vascular inflammation (Fold = 2.94, FDR = 0.3142), which potentially reflects underlying vascular complications (32); and Circadian rhythm (Fold = 2.67, FDR = 0.2930), which is increasingly recognized as a contributor to metabolic dysregulation (33).

Among the miRNAs, members of the hsa-miR-124 family (hsa-miR-124-1, hsa-miR-124-2, hsa-miR-124-3) and hsa-miR-125b (hsa-miR-125b-1, hsa-miR-125b-2) appeared in multiple enriched categories, suggesting central regulatory roles in both metabolic and immunological pathways. These miRNAs may represent promising candidates for future functional studies, pending experimental validation, rather than established biomarkers (6, 34).

Collectively, the enrichment analysis supports the hypothesis that miRNA dysregulation in diabetes reflects not only impaired metabolic processes but also broader disruptions in immune regulation, cellular differentiation, and systemic homeostasis. Pathways or categories with FDR > 0.2 were treated as nominal enrichments and are interpreted cautiously.

From a disease perspective, differentially expressed miRNAs were significantly associated with multiple diabetes-related pathologies. Notably, diabetic nephropathy (FDR = 0.0171), acute myocardial infarction (FDR = 0.0211), tuberculosis (pulmonary) (FDR = 0.0234), rhinosinusitis (FDR = 0.0175), Machado-Joseph disease (FDR = 6.24e-4), and myelodysplastic syndromes (FDR = 2.39e-3) showed strong enrichment. Several other diseases with strong associations (all FDR < 0.05) included: Neuroinflammation, epilepsy, Alzheimer’s disease, acute kidney injury, stroke, HIV infection, and Hepatitis C virus infection.

Interestingly, although Type 2 diabetes mellitus (T2DM) was present in the dataset, its false discovery rate (FDR) exceeded the significance threshold (FDR = 1). This suggests that the differentially expressed miRNAs may serve as more reliable indicators of diabetic complications than of the disease itself.

Several microRNAs (miRNAs) have been consistently implicated in various diseases, particularly members of the hsa-miR-124, hsa-miR-125b, hsa-miR-181a, hsa-miR-221, and hsa-miR-194 families. These miRNAs create a core regulatory signature associated with inflammatory and metabolic disorders, different malignancies, and neurodegenerative conditions. The presence of these miRNAs in diverse yet pathophysiologically interconnected diseases indicates a broader role for miRNA-mediated regulation in the systemic manifestations of diabetes.

TAM 2.0 tissue enrichment analysis of miRNAs differentially expressed between prediabetic and non-diabetic individuals revealed significant associations with various tissues. Notably, the eye showed the strongest enrichment (FDR = 0.0262), with associated miRNAs including hsa-miR-182, hsa-miR-3183, and hsa-miR-7158, suggesting early retinal or neuro-ocular involvement, even before the onset of clinical diabetes. Additional tissues with significant enrichment included the artery (FDR = 0.1006), stomach (FDR = 0.1207), bone (FDR = 0.2056), adipose tissue (FDR = 0.2056), and smooth muscle (FDR = 0.1926).

Functional annotation of miRNAs differing between prediabetic and non-diabetic plasma samples revealed enrichment in several biological processes relevant to metabolic dysfunction, immune regulation, and cellular differentiation. The top enriched functions (FDR < 0.05) included Glucose Metabolism (FDR = 0.0188), highlighting the strong association of these miRNAs with early dysregulation in metabolic pathways, Cell Differentiation (FDR = 0.0401) and Inflammation (FDR = 0.0489), both critical in the pathogenesis of insulin resistance and prediabetes, and Neuron Differentiation (FDR = 0.0492) and Regulation of Stem Cell Functions (FDR = 0.0545), suggesting possible neuroendocrine and regenerative component alterations in prediabetic individuals.

The TAM 2.0 enrichment analysis of miRNAs differentially expressed between prediabetic and non-diabetic individuals identified significant associations with several disease categories. The most significantly enriched disease terms included Diabetic Nephropathy (FDR = 8.18 × 10^-^³), Rhinosinusitis (FDR = 7.23 × 10^-^³), Myelodysplastic Syndromes (FDR = 0.0188), Acute Kidney Injury (FDR = 0.0262), and Medulloblastoma (FDR = 0.0281). Additional terms with strong enrichment included Toxic Epidermal Necrolysis, Stroke, Hemorrhagic, Early-Stage Colon Carcinoma, and Cervical Neoplasms, all with FDR < 0.05. A set of core miRNAs, hsa-miR-124-2, hsa-miR-124-1, hsa-miR-124-3, hsa-miR-125 b-1, hsa-miR-125 b-2, and hsa-miR-135a-2 were recurrently implicated across a range of enriched disease phenotypes, particularly in cancer, neurological, and inflammatory contexts.

Compared to the diabetic group, the prediabetic group exhibited a broader and more diverse disease enrichment profile that includes cancers (e.g., pancreatic, colon, prostate), neuroinflammatory, and autoimmune diseases. It also showed stronger associations with neoplasms and immune-related conditions, possibly reflecting early dysregulation before the onset of overt hyperglycemia. The prediabetic group shared miRNA signatures with the diabetic group, indicating early activation of pathological pathways (notably hsa-miR-124 and hsa-miR-125b families), while also possessing unique signatures in prediabetes that may suggest distinct regulatory responses.

### Differential expression of miRNAs by sex

To gain a deeper understanding of the molecular mechanisms underlying sex differences in diabetes progression, we comprehensively analyzed circulating miRNA expression across various glycemic states, taking into account sex. Considering the established biological and clinical disparities between males and females in metabolic diseases, our approach aimed to delineate miRNA signatures associated with sex, diabetes, and prediabetes. We first assessed sex-based differences in miRNA expression independent of disease status, followed by targeted comparisons within diabetic males and females. Subsequently, we examined differential expression patterns between diabetic and control groups, as well as between prediabetic and control groups, with sex included as a covariate. This stratified analysis facilitated the identification of both shared and unique miRNA signatures associated with glucose dysregulation (**Supplementary Table S3**). **Supplementary Table S3** provides the complete list of miRNAs differentially expressed by sex and glycemic status, including their log_2_ fold changes and FDR values.

### miRNA expression differences between males and females independent of disease status

To investigate sex-specific differences in miRNA expression, regardless of disease status, we conducted a differential expression analysis comparing all male participants to all female participants. This analysis revealed a set of miRNAs significantly upregulated in males, including hsa-miR-4289 (log_2_FC = 2.41, FDR = 9.78 × 10^-5^), hsa-miR-4432 (log_2_FC = 2.59, FDR = 0.0016), and hsa-miR-514a-5p (log_2_FC = 1.70, FDR = 0.0036), each demonstrating strong statistical significance (uncorrected p < 5 × 10^-6^). All values were adjusted for multiple testing, and uncorrected p-values are reported for transparency but interpreted as exploratory. These results suggest substantial sex-based differences in the expression of specific miRNAs, which may contribute to the underlying biological and pathophysiological disparities observed between males and females in diabetes and related metabolic disorders. Additional miRNAs with moderate fold changes but high statistical significance were also identified, indicating a broader regulatory divergence by sex that warrants further investigation.

### Differential miRNA expression in diabetic males versus diabetic females

Next-generation sequencing identified several miRNAs that were differentially expressed between diabetic males and females. Notably, hsa-miR-4289 and hsa-miR-4432 were significantly downregulated in males compared to females, with log_2_ fold changes of -2.50 and -2.94, respectively, and false discovery rates (FDRs) of 0.011. These findings suggest potential sex-specific regulatory roles of miRNAs in diabetes. Additional miRNAs, such as hsa-miR-3143 and hsa-miR-514a-5p, also displayed trends of downregulation in males, albeit with higher FDR values. In contrast, hsa-miR-520e-3p was upregulated in males (log_2_ fold change = 2.19); however, this change did not achieve statistical significance after FDR correction.

### Differential miRNA expression in diabetic versus control subjects accounting for sex

Analysis of miRNA expression profiles between diabetic and control individuals, adjusted for sex, revealed 40 miRNAs with significant differential expression (FDR < 0.05). Among the most upregulated in diabetes were hsa-let-7i-5p (log_2_FC = 2.52), hsa-miR-221-3p (log_2_FC = 2.47), hsa-let-7d-5p (log_2_FC = 2.21), and hsa-miR-126-3p (log_2_FC = 2.18), suggesting their potential involvement in diabetes-related pathways. Conversely, miRNAs such as hsa-miR-6751-3p (log_2_FC = -2.47), hsa-miR-520e-3p (log_2_FC = -1.41), and hsa-miR-196b-3p (log_2_FC = -1.17) were significantly downregulated in diabetic individuals. These findings indicate a broad miRNA signature associated with diabetes that is resilient to sex differences, highlighting candidates for further investigation as potential biomarkers or therapeutic targets.

Enrichment analysis showed that significant miRNAs were enriched in diabetes-related biological processes. Major functions included cell proliferation (13 miRNAs, FDR = 7.01 × 10^-8^), hematopoiesis (10 miRNAs, FDR = 1.86 × 10^-6^), and inflammation (13 miRNAs, FDR = 3.77 × 10^-6^). Other pathways enriched were stem cell regulation (FDR = 3.01 × 10^-5^), apoptosis (FDR = 0.0294), and angiogenesis (FDR = 0.0461). Several diabetes-related pathways were also enriched, including glucose metabolism (FDR = 0.0143), insulin resistance, vascular inflammation (FDR = 2.61 × 10^-4^), and immune response (FDR = 1.98 × 10^-5^). Disease association analysis revealed significant enrichment for type 2 diabetes mellitus (FDR = 0.0451), diabetic retinopathy, and diabetic vasculopathy, as well as cardiovascular conditions such as acute myocardial infarction (FDR = 6.21 × 10^-4^) and coronary heart disease (FDR = 3.87 × 10^-4^). Additionally, the miRNAs were enriched in various neoplastic, autoimmune, and neurodegenerative disease pathways, indicating broader systemic involvement. Tissue and cell type specificity analysis revealed that differentially expressed miRNAs were predominantly found in vascular-associated and immune-related cells, including endothelial cells, renal epithelial cells, monocytes, and hematopoietic progenitor cells. Overall, these findings suggest that the identified miRNAs play a role in regulating inflammation, vascular remodeling, and metabolic homeostasis in diabetes.

### Differential miRNA expression in prediabetic versus control subjects accounting for sex

Differential expression analysis of miRNAs between prediabetic and control individuals, adjusted for sex, revealed seven with statistically significant differences (FDR < 0.05). Among these, hsa-miR-4800-5p, hsa-miR-4693-3p, hsa-miR-5002-3p, hsa-miR-3661, and hsa-miR-4429 were significantly upregulated in the prediabetic group, with log_2_ fold changes ranging from 0.84 to 2.87. In contrast, hsa-miR-1277-5p and hsa-miR-1204 were significantly downregulated (log_2_ fold changes of -2.22 and -1.04, respectively). These sex-adjusted results suggest a panel of miRNAs that may reflect early regulatory changes associated with the transition from normoglycemia to prediabetes.

Enrichment analysis revealed distinct associations across disease, tissue, and cell-type categories. Among disease terms, acute ischemic stroke exhibited the most substantial enrichment, particularly with hsa-miR-4429, which was upregulated in the dataset (fold enrichment = 58.5, Bonferroni-adjusted p = 0.002). At the cell-type level, particular enrichment was noted for hsa-miR-4800, omental adipocytes (hsa-miR-4693), and retinal pigment epithelial cells (hsa-miR-3661 and hsa-miR-5002), with fold enrichments exceeding 17 and Bonferroni-adjusted p-values < 0.01. Additional enrichment in gingival epithelial cells, mast cells, and neural stem cells suggests involvement of various biological systems. These findings highlight the potential functional significance of sex-biased miRNAs in the transition from prediabetes to diabetes, particularly in vascular, metabolic, and neural contexts.

### Aspirin effect differential analysis

**Supplementary Table S4** summarizes all miRNAs assessed for aspirin-associated differential expression across diabetic subgroups, including fold-change values and corresponding p- and FDR-adjusted significance levels.

### Differential miRNA expression in diabetic individuals taking versus not taking aspirin

To investigate the potential influence of aspirin intake on circulating miRNA profiles in individuals with diabetes, we conducted a differential expression analysis comparing diabetic patients who took aspirin with those who did not receive aspirin therapy. While several miRNAs showed moderate fold changes and strong unadjusted p-values, none passed the threshold for statistical significance after correction for multiple testing (FDR < 0.05). Among the top-ranked candidates based on unadjusted p-values were hsa-miR-4263 (log_2_FC = 1.03, unadjusted p = 1.12 × 10^-4^), hsa-miR-548s (log_2_FC = 0.98, p = 3.75 × 10^-4^), and hsa-miR-204-5p (log_2_FC = 1.35, p = 6.01 × 10^-4^), all of which were upregulated in the aspirin group. Despite these trends, none remained statistically significant after FDR adjustment, suggesting that while aspirin may influence the expression of specific miRNAs, these effects are subtle or variable in this cohort. Although none of the differences reached statistical significance after FDR correction, these unadjusted results are reported as exploratory and should be interpreted as trend-level findings. Further studies with larger sample sizes may help clarify the regulatory impact of aspirin on miRNA expression in diabetes.

### Differential miRNA expression in diabetic patients not taking aspirin versus non-diabetic controls

To evaluate miRNA expression differences associated with diabetes independent of aspirin therapy, we compared diabetic patients not taking aspirin with non-diabetic control individuals. This analysis identified only three significantly differentially expressed miRNAs. Notably, hsa-miR-4708-5p showed the strongest downregulation in the diabetic group (log_2_ fold change = –0.54; FDR = 2.27 × 10^-4^), along with hsa-miR-4482-5p and hsa-miR-152-5p, both of which were also downregulated with FDR values < 0.05. Additional miRNAs such as hsa-miR-4708-3p and hsa-miR-1197 displayed similar downregulation patterns, although their FDR values were above the significance threshold. These patterns are therefore described as exploratory and not statistically confirmed. These findings suggest that specific miRNAs may be potential markers of diabetes-related dysregulation, independent of anti-inflammatory drug influence. The consistent downregulation of miRNAs involved in regulatory and metabolic processes underscores their potential role in the pathophysiology of diabetes.

### Differential miRNA expression in diabetic patients taking aspirin versus non-diabetic controls

To determine whether aspirin use impacts miRNA expression patterns in diabetic individuals compared to non-diabetic controls, we analyzed circulating miRNA profiles in diabetic patients undergoing aspirin therapy versus healthy controls. This comparison revealed several miRNAs with significant differential expression. Notably, hsa-miR-4708-5p was the most significantly downregulated miRNA in the aspirin-treated diabetic group (log_2_ fold change = –0.61; FDR = 6.2 × 10^-5^). Additionally, miRNAs such as hsa-miR-6513-5p and hsa-miR-20b-3p were significantly upregulated (log_2_FC = 0.75 and 1.22; FDR = 0.016 and 0.020, respectively). Other miRNAs, including hsa-miR-8054 and hsa-miR-3149, were downregulated and reached statistical significance (FDR = 0.020). These findings suggest that aspirin therapy may lead to specific changes in miRNA expression in diabetic patients compared to healthy controls, with implications for anti-inflammatory or metabolic regulatory mechanisms.

Although several miRNAs showed nominal significance, none remained significant after multiple-testing correction. These observations represent exploratory trends rather than definitive differences and illustrate the potential modifying role of medications, which should be investigated in larger, independent cohorts.

### Correlation analysis

**Supplementary Table S5** summarizes the complete correlation matrix for all biochemical and clinical traits across the study population and within sex- and disease-specific subgroups.

### Correlations among clinical and biochemical traits

**Figure 2:**
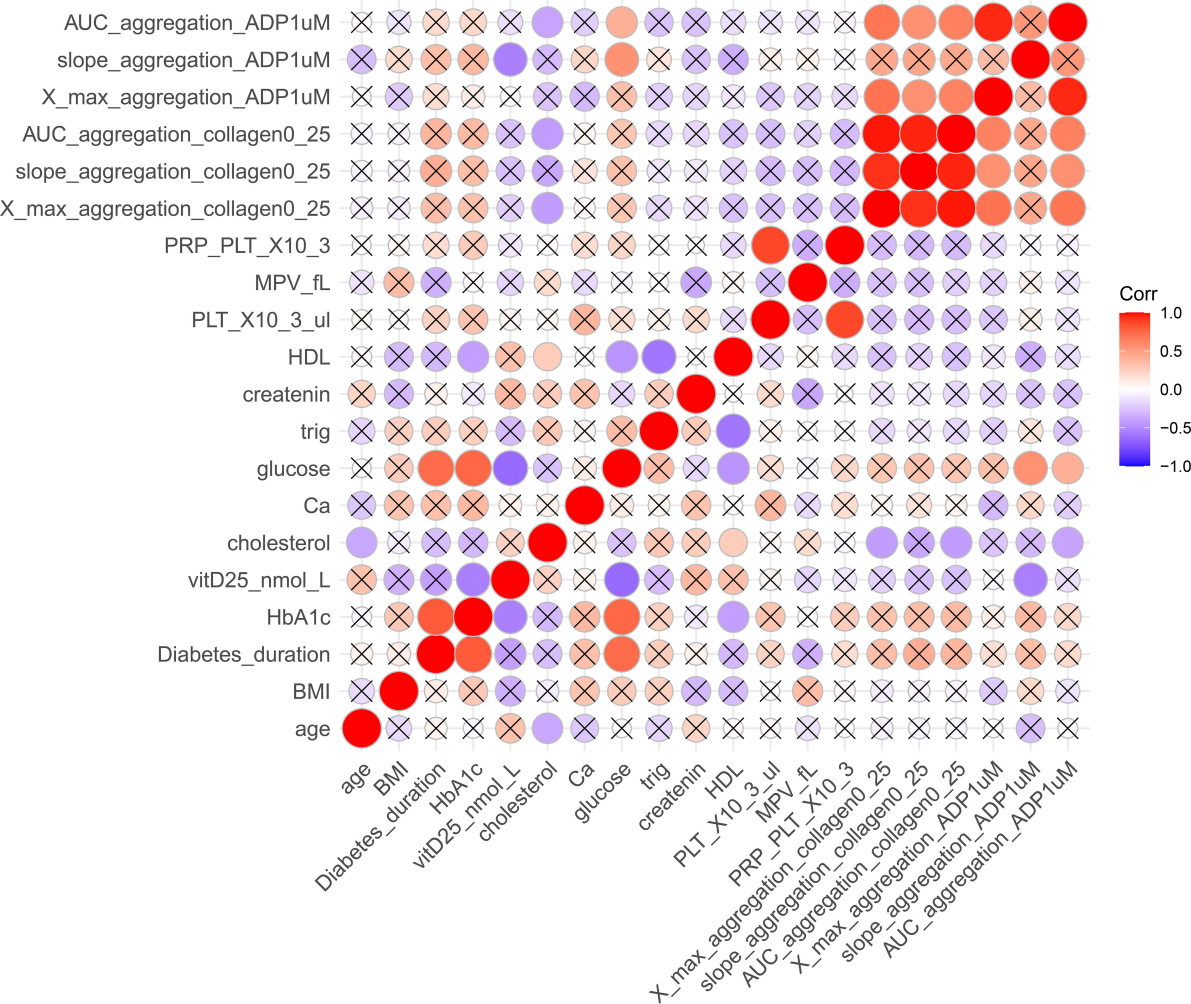
Correlation matrix of clinical, biochemical, and platelet function variables. The heatmap depicts Pearson correlation coefficients among selected traits. Colors range from red (strong positive correlation, r = +1) to blue (strong negative correlation, r = –1). Only statistically significant correlations (p < 0.05) are displayed as colored circles, while non-significant correlations are marked with an “×”. Circle size is proportional to correlation strength. AUC_aggregation_ADP1uM, area under the curve for adenosine diphosphate (1 μM)–induced aggregation; slope_aggregation_ADP1uM, aggregation slope for ADP (1 μM); X_max_aggregation_ADP1uM, maximum aggregation for ADP (1 μM); AUC_aggregation_collagen0_25, area under the curve for collagen (0.25 μg/mL)–induced aggregation; slope_aggregation_collagen0_25, aggregation slope for collagen (0.25 μg/mL); X_max_aggregation_collagen0_25, maximum aggregation for collagen (0.25 μg/mL); MPV_fL, mean platelet volume (fL); PRP_PLT_X10^3^, platelet-rich plasma platelet count (×10³/μL); HDL, high-density lipoprotein cholesterol; creatinine, serum creatinine; trig, triglycerides; glucose, fasting blood glucose; Ca, calcium; cholesterol, total cholesterol; vitD25, 25-hydroxyvitamin D (nmol/L); HbA1c, glycated hemoglobin; Diabetes_duration, years since diagnosis; BMI, body mass index.

Pearson correlation analyses were performed across the entire study population (**Figure 2**) and within subgroups (**Supplementary Table S5**) defined by disease status and sex to assess the relationships between clinical and laboratory phenotypic variables. In the overall group, strong positive correlations were noted between whole blood platelet count (PLT_X10³/μL) and platelet-rich plasma platelet count (PRP_PLT_X10³) (r = 0.86, p = 5.88 × 10^-8^), diabetes duration and HbA1c (r = 0.82, p = 1.41 × 10^-6^), and HbA1c and glucose levels (r = 0.78, p = 2.03 × 10^-5^), indicating consistently strong links between glycemic exposure and current metabolic status. Inversely, vitamin D levels (vitD25_nmol_L) correlated with glucose (r = –0.52, p = 0.013), HbA1c (r = –0.45, p = 0.034), and platelet aggregation response (ADP1_agg_slope, r = –0.49, p = 0.021), suggesting a relationship between lower vitamin D status, glycemic control, and platelet activity as described previously by our lab at Sultan et al., 2018. Subgroup analyses demonstrated similar patterns, in which sex-stratified analyses revealed significant correlations between platelet parameters in both females (r = 0.82) and males (r = 0.89), along with additional correlations between diabetes duration and glucose (r = 0.68), and HbA1c (r=0.81) in males, and in females (r=0.8). In females, vitamin D levels positively correlated with serum calcium (r = 0.61, p = 0.045) and creatinine (r = 0.64, p = 0.034), whereas in males, vitamin D was inversely associated with platelet aggregation (r = –0.75, p = 0.008). These results highlight potential metabolic and hemostatic functions of vitamin D across different sex and disease categories.

### Correlations between phenotypic traits and circulating hsa-miRs

**Figure 3:**
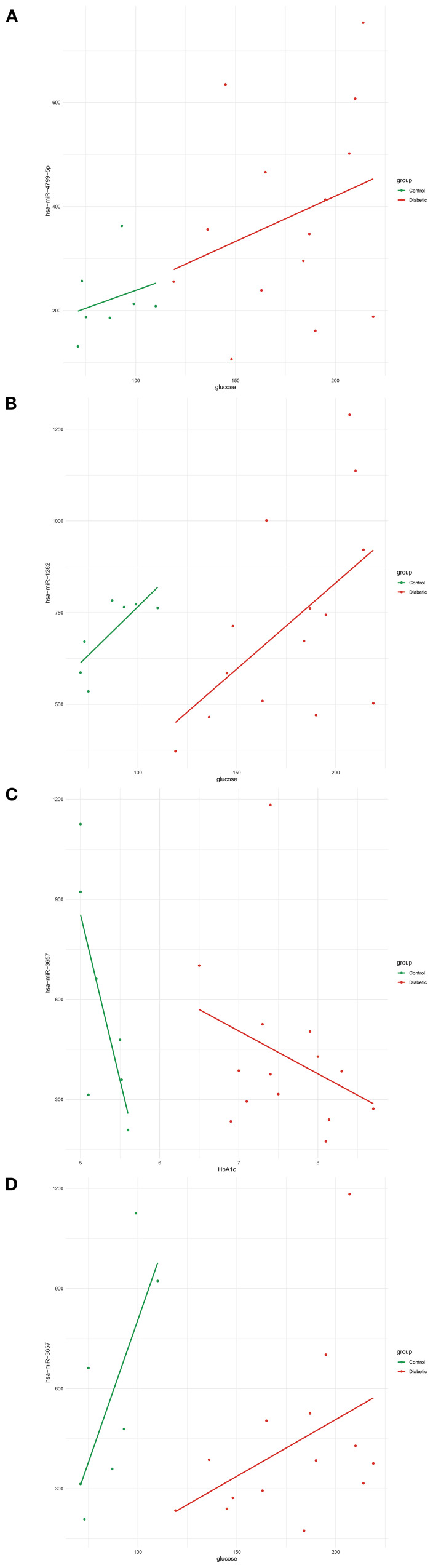
Correlations between circulating hsa-miRs and glucose-regulation–related phenotypic traits. **(A)** Positive correlation between hsa-miR-4799-5p expression and fasting glucose levels (mg/dL) in diabetic patients compared to controls. **(B)** Positive correlation between hsa-miR-1282 expression and fasting glucose levels (mg/dL). **(C)** Negative correlation between hsa-miR-3657 expression and HbA1c (%), reflecting long-term glycemic control. **(D)** Positive correlation between hsa-miR-3657 expression and fasting glucose levels (mg/dL), reflecting short-term glycemic status. Lines represent linear regression fits (green, control group; red, diabetic group). Abbreviations: hsa-miR, human microRNA; HbA1c, glycated hemoglobin.

All correlation analyses are descriptive and exploratory; reported associations do not imply causality. Several miRNAs exhibited notable associations with metabolic traits, particularly those related to glucose. Among them, hsa-miR-4799-5p displayed a glucose correlation pattern influenced by diabetes status (**Figure 3A**), whereas hsa-miR-1282 correlated with glucose levels independent of diabetes (**Figure 3B**). Additionally, hsa-miR-3657 revealed contrasting relationships with glycemic markers: it was positively correlated with HbA1c, an indicator of long-term glycemic control (**Figure 3C**), but negatively correlated with glucose, reflecting short-term glycemia (**Figure 3D**). Despite the strong relationships between vitamin D and metabolic measures, no significant correlations were identified between miRNAs and vitamin D (vitD25_nmol_L) levels in this dataset. These results underscore the complexity of miRNA–phenotype interactions and suggest that specific miRNAs may play distinct roles in regulating short- and long-term glycemic control, potentially affecting the molecular pathophysiology of diabetes.

## Discussion

This study provides a comprehensive analysis of circulating microRNA (miRNA) profiles in diabetic, pre-diabetic, and healthy individuals, utilizing next-generation sequencing (NGS) to uncover differentially expressed miRNAs and their associations with key metabolic, inflammatory, and vascular phenotypes (16, 35). Importantly, our work encompasses various layers of analysis, including disease status, sex, vitamin D levels, platelet function, and aspirin use, thereby providing a multifaceted perspective on miRNA regulation in diabetes pathophysiology (36, 37).

This study is particularly unique in its integration of glycemic control, vitamin D status, and platelet function with miRNA expression patterns, a multidimensional approach not commonly applied in previous studies (38, 39). Additionally, our subgroup analyses, which compare diabetic patients who use aspirin with those who do not, along with sex-specific miRNA patterns, offer essential insights into the molecular diversity of diabetes that are frequently neglected (40–42). The aspirin-related comparisons in this study were exploratory, as no differences remained significant after multiple-testing correction, and should therefore be interpreted as trend-level observations pending validation in larger cohorts.

This study identified several miRNAs, particularly hsa-miR-4799-5p and hsa-miR-3657, showing distinct correlations with glycemic levels. These findings highlight the complex roles of miRNAs in regulating glucose homeostasis in both short and long terms. Previous research by *Li et al.* (2020) noted hsa-miR-4799-5p in a hypertension-related co-expression network with indirect links to insulin signaling (43). Our study suggests a potential direct association between this miRNA and glucose levels, indicating its broader role in metabolic regulation, while this observation should be interpreted as exploratory rather than a confirmed functional role. While hsa-miR-4799-5p demonstrated a modest increase in individuals with diabetes compared to controls (log2FC = 0.76, p = 0.0127), it did not achieve statistical significance after false discovery rate (FDR) correction (FDR = 0.123). Therefore, this observation is considered a trend-level finding that requires validation in larger datasets. Nonetheless, its robust association with glycemic traits suggests a potential functional role in metabolic regulation that differential expression analysis may not adequately address.

Similarly, hsa-miR-3657 shows a negative correlation with fasting glucose and a positive correlation with HbA1c, indicating varying responses to hyperglycemia. To our knowledge, this is the first study suggesting an association between hsa-miR-3657 and metabolic traits, indicating its potential role in transitioning from acute (glucose levels) to chronic (HbA1C) glycemic dysregulation. Further studies are needed to clarify the roles of these miRNAs in metabolic tissues and evaluate their potential as candidate biomarkers or therapeutic targets for type 2 diabetes.

A key strength is its high-throughput, unbiased miRNA profiling using NGS, which allows for the detection of thousands of circulating miRNAs simultaneously (44, 45). Additionally, combining multiple bioinformatics tools, including enrichment analyses via TAM 2.0, helps identify potential biological pathways and tissue associations related to early diabetic changes, such as vascular remodeling, inflammation, and immune responses (26).

Given the exploratory nature of this research, future studies should aim to validate the most promising miRNAs, such as hsa-miR-4776-5p, hsa-miR-5002-3p, and hsa-miR-4800-5p, using quantitative PCR (qPCR) in larger, well-characterized cohorts. These investigations should include longitudinal sampling to monitor how miRNA expression changes during the progression and treatment of diabetes. In particular, expanding the pre-diabetic group is crucial for understanding the early miRNA alterations that occur before hyperglycemia appears, which could aid in the development of new early diagnosis and prevention strategies (49).

## Limitations

This study has several limitations. The cohort size was small (n = 24), particularly in the pre-diabetic group (n = 2), which limits statistical power and generalizability. Therefore, the findings should be viewed as exploratory, although the overlap with literature-validated miRNAs strengthens confidence in their relevance (46, 47).

This study has several limitations. The cohort size was small (n = 24), particularly in the pre-diabetic group (n = 2), which substantially reduces statistical power and the ability to generalize results. The findings should therefore be interpreted as exploratory and hypothesis-generating rather than confirmatory, although the overlap with literature-validated miRNAs strengthens confidence in their relevance (46, 47).

The plasma samples were originally collected for the Sultan et al. (2019) study (22), which focused on platelet aggregation, vitamin D, and glycemic control but did not assess microRNAs. The novelty of our work lies in applying next-generation sequencing to this archived cohort, thereby expanding the scope of the dataset. During the COVID-19 pandemic, research laboratory activity was suspended, and by the time NGS analysis resumed, several archived pre-diabetic samples did not meet quality control standards, leaving only two suitable for inclusion. All plasma samples were stored at −80 °C and subjected to a single thaw cycle prior to RNA extraction; however, long-term storage and freeze–thaw may affect RNA integrity and are acknowledged as potential limitations. In addition, all RNA samples underwent quality-control assessment before sequencing, and any that did not meet QC standards were excluded from analysis. Potential batch effects related to sequencing runs or library preparation cannot be entirely ruled out, although identical protocols and simultaneous processing were applied to minimize variability. Moreover, the study did not include downstream functional or mechanistic validation, which remains an important next step.

Finally, while we recorded clinical variables (age, sex, BMI, comorbidities, medications) and performed stratified analyses, residual confounding cannot be excluded. Unmeasured lifestyle factors such as diet and physical activity, as well as other medications, may have influenced results and should be considered in future studies. Moreover, the associations observed between miRNAs and platelet aggregation, vitamin D, and HbA1c are correlational rather than causal and require validation in larger, prospective studies (48). Similarly, all correlation analyses were descriptive and intended to highlight potential relationships rather than infer causation.

In summary, our study highlights circulating microRNAs as candidate molecular signals involved in the regulation of glycemic control and diabetes-associated vascular processes. Several of the identified miRNAs, including those with consistent associations across glycemic traits and published datasets, may represent exploratory indicators of metabolic dysregulation. By integrating miRNA profiles with biochemical traits, platelet activity, and sex-specific differences, this work provides a foundation for future validation studies aimed at developing more personalized approaches to diabetes risk assessment (42, 49).

## Data Availability

The raw sequencing data are in preparation for deposition in the NCBI Gene Expression Omnibus (GEO) repository. The accession number will be provided once the upload process is completed. Processed count data and relevant metadata are available from the corresponding author upon reasonable request.

## References

[1] International Diabetes Federation . IDF Diabetes Atlas (2021). International Diabetes Federation. Available online at: https://diabetesatlas.org/ (Accessed October 20, 2025).

[2] Sun H Saeedi P Karuranga S Pinkepank M Ogurtsova K Duncan BB . Erratum to “IDF Diabetes Atlas: Global, regional and country-level diabetes prevalence estimates for 2021 and projections for 2045” [Diabetes Res. Clin. Pract. 183 (2022) 109119. Diabetes Res Clin Pract. (2023) 204:110945. doi: 10.1016/j.diabres.2023.110945, PMID: 37863776

[3] Sun H Saeedi P Karuranga S Pinkepank M Ogurtsova K Duncan BB . IDF Diabetes Atlas: Global, regional and country-level diabetes prevalence estimates for 2021 and projections for 2045. Diabetes Res Clin Pract. (2022) 183:109119. doi: 10.1016/j.diabres.2021.109119, PMID: 34879977 PMC11057359

[4] Antar SA Ashour NA Sharaky M Khattab M Ashour NA Zaid RT . Diabetes mellitus: Classification, mediators, and complications; A gate to identify potential targets for the development of new effective treatments. BioMed Pharmacother. (2023) 168:115734. doi: 10.1016/j.biopha.2023.115734, PMID: 37857245

[5] Kaur G Lakshmi PVM Rastogi A Bhansali A Jain S Teerawattananon Y . Diagnostic accuracy of tests for type 2 diabetes and prediabetes: A systematic review and meta-analysis. PloS One. (2020) 15:e0242415. doi: 10.1371/journal.pone.0242415, PMID: 33216783 PMC7678987

[6] Bergman M Abdul-Ghani M DeFronzo RA Manco M Sesti G Fiorentino TV . Review of methods for detecting glycemic disorders. Diabetes Res Clin Pract. (2020) 165:108233. doi: 10.1016/j.diabres.2020.108233, PMID: 32497744 PMC7977482

[7] Bartel DP . MicroRNAs. Cell. (2004) 116:281–97. doi: 10.1016/S0092-8674(04)00045-5, PMID: 14744438

[8] Sou YL Chilian WM Ratnam W Zain SM Syed Abdul Kadir SZ Pan Y . Exosomal miRNAs and isomiRs: potential biomarkers for type 2 diabetes mellitus. Precis Clin Med. (2024) 7:pbae021. doi: 10.1093/pcmedi/pbae021, PMID: 39347441 PMC11438237

[9] Li Y Kowdley KV . MicroRNAs in common human diseases. Genomics Proteomics Bioinf. (2012) 10:246–53. doi: 10.1016/j.gpb.2012.07.005, PMID: 23200134 PMC3611977

[10] Kristensen LS Andersen MS Stagsted LVW Ebbesen KK Hansen TB Kjems J . The biogenesis, biology and characterization of circular RNAs. Nat Rev Genet. (2019) 20:675–91. doi: 10.1038/s41576-019-0158-7, PMID: 31395983

[11] Angelescu MA Andronic O Dima SO Popescu I Meivar-Levy I Ferber S . miRNAs as biomarkers in diabetes: moving towards precision medicine. Int J Mol Sci. (2022) 23:12843. doi: 10.3390/ijms232112843, PMID: 36361633 PMC9655971

[12] Kozłowska M Śliwińska A . The link between diabetes, pancreatic tumors, and miRNAs—New players for diagnosis and therapy? Int J Mol Sci. (2023) 24:10252. doi: 10.3390/ijms241210252, PMID: 37373398 PMC10299694

[13] Wicik Z Czajka P Eyileten C Fitas A Wolska M Jakubik D . The role of miRNAs in regulation of platelet activity and related diseases - a bioinformatic analysis. Platelets. (2022) 33:1052–64. doi: 10.1080/09537104.2022.2042233, PMID: 35285386

[14] Vasu S Kumano K Darden CM Rahman I Lawrence MC Naziruddin B . MicroRNA signatures as future biomarkers for diagnosis of diabetes states. Cells. (2019) 8:1533. doi: 10.3390/cells8121533, PMID: 31795194 PMC6953078

[15] Aryani A Denecke B . *In vitro* application of ribonucleases: comparison of the effects on mRNA and miRNA stability. BMC Res Notes. (2015) 8:164. doi: 10.1186/s13104-015-1114-z, PMID: 25899823 PMC4411928

[16] Guay C Regazzi R . Circulating microRNAs as novel biomarkers for diabetes mellitus. Nat Rev Endocrinol. (2013) 9:513–21. doi: 10.1038/nrendo.2013.86, PMID: 23629540

[17] Nunez Lopez YO Garufi G Seyhan AA . Altered levels of circulating cytokines and microRNAs in lean and obese individuals with prediabetes and type 2 diabetes. Mol Biosyst. (2017) 13:106–21. doi: 10.1039/C6MB00596A, PMID: 27869909

[18] Hu F Liu L Liu Z Cao M Li G Zhang X . Meta-analysis of the characteristic expression of circulating microRNA in type 2 diabetes mellitus with acute ischemic cerebrovascular disease. Front Endocrinol. (2023) 14:1129860. doi: 10.3389/fendo.2023.1129860, PMID: 36864836 PMC9971585

[19] Yan S Wang T Huang S Di Y Huang Y Liu X . Differential expression of microRNAs in plasma of patients with prediabetes and newly diagnosed type 2 diabetes. Acta Diabetol. (2016) 53:693–702. doi: 10.1007/s00592-016-0837-1, PMID: 27039347

[20] Zhu H Leung Sw . MicroRNA biomarkers of type 2 diabetes: evidence synthesis from meta-analyses and pathway modelling. Diabetologia. (2023) 66:288–99. doi: 10.1007/s00125-022-05809-z, PMID: 36269347 PMC9807484

[21] Paseban M Marjaneh RM Banach M Riahi MM Bo S Sahebkar A . Modulation of microRNAs by aspirin in cardiovascular disease. Trends Cardiovasc Med. (2020) 30:249–54. doi: 10.1016/j.tcm.2019.08.005, PMID: 31444100

[22] Sultan M Twito O Tohami T Ramati E Neumark E Rashid G . Vitamin D diminishes the high platelet aggregation of type 2 diabetes mellitus patients. Platelets. (2019) 30:120–5. doi: 10.1080/09537104.2017.1386298, PMID: 29313404

[23] Ewels PA Peltzer A Fillinger S Patel H Alneberg J Wilm A . The nf-core framework for community-curated bioinformatics pipelines. Nat Biotechnol. (2020) 38:276–8. doi: 10.1038/s41587-020-0439-x, PMID: 32055031

[24] Chen S Zhou Y Chen Y Gu J . fastp: an ultra-fast all-in-one FASTQ preprocessor. Bioinformatics. (2018) 34:i884–90. doi: 10.1093/bioinformatics/bty560, PMID: 30423086 PMC6129281

[25] Love MI Huber W Anders S . Moderated estimation of fold change and dispersion for RNA-seq data with DESeq2. Genome Biol. (2014) 15:550. doi: 10.1186/s13059-014-0550-8, PMID: 25516281 PMC4302049

[26] Li J Han X Wan Y Zhang S Zhao Y Fan R . TAM 2.0: tool for MicroRNA set analysis. Nucleic Acids Res. (2018) 46:W180–5. doi: 10.1093/nar/gky509, PMID: 29878154 PMC6031048

[27] Lu M Shi B Wang J Cao Q Cui Q . TAM: A method for enrichment and depletion analysis of a microRNA category in a list of microRNAs. BMC Bioinf. (2010) 11:419. doi: 10.1186/1471-2105-11-419, PMID: 20696049 PMC2924873

[28] Berry C Lal M Binukumar BK . Crosstalk between the unfolded protein response, microRNAs, and insulin signaling pathways: in search of biomarkers for the diagnosis and treatment of type 2 diabetes. Front Endocrinol. (2018) 9:210. doi: 10.3389/fendo.2018.00210, PMID: 29770126 PMC5940743

[29] Tao L Liu H Gong Y . Role and mechanism of the Th17/Treg cell balance in the development and progression of insulin resistance. Mol Cell Biochem. (2019) 459:183–8. doi: 10.1007/s11010-019-03561-4, PMID: 31218568 PMC6679830

[30] Purvis N Kumari S Chandrasekera D Bellae Papannarao J Gandhi S Van Hout I . Diabetes induces dysregulation of microRNAs associated with survival, proliferation and self-renewal in cardiac progenitor cells. Diabetologia. (2021) 64:1422–35. doi: 10.1007/s00125-021-05405-7, PMID: 33655378

[31] Hofbauer LC Busse B Eastell R Ferrari S Frost M Müller R . Bone fragility in diabetes: novel concepts and clinical implications. Lancet Diabetes Endocrinol. (2022) 10:207–20. doi: 10.1016/S2213-8587(21)00347-8, PMID: 35101185

[32] Abdel Mageed SS Doghish AS Ismail A El-Husseiny AA Fawzi SF Mahmoud AMA . The role of miRNAs in insulin resistance and diabetic macrovascular complications – A review. Int J Biol Macromol. (2023) 230:123189. doi: 10.1016/j.ijbiomac.2023.123189, PMID: 36623613

[33] Du N Sinturel F Nowak N Gosselin P Saini C Guessous I . Multi-omics correlates of insulin resistance and circadian parameters mapped directly from human serum. Eur J Neurosci. (2024) 60:5487–504. doi: 10.1111/ejn.16486, PMID: 39205434

[34] Cheung R Pizza G Chabosseau P Rolando D Tomas A Burgoyne T . Glucose-dependent miR-125b is a negative regulator of β-cell function. Diabetes. (2022) 71:1525–45. doi: 10.2337/db21-0803, PMID: 35476777 PMC9998846

[35] Wang J Chen J Sen S . MicroRNA as biomarkers and diagnostics. J Cell Physiol. (2016) 231:25–30. doi: 10.1002/jcp.25056, PMID: 26031493 PMC8776330

[36] Pescador N Pérez-Barba M Ibarra JM Corbatón A Martínez-Larrad MT Serrano-Ríos M . Serum circulating microRNA profiling for identification of potential type 2 diabetes and obesity biomarkers. PloS One. (2013) 8:e77251. doi: 10.1371/journal.pone.0077251, PMID: 24204780 PMC3817315

[37] He X Kuang G Wu Y Ou C . Emerging roles of exosomal miRNAs in diabetes mellitus. Clin Transl Med. (2021) 11:e468. doi: 10.1002/ctm2.468, PMID: 34185424 PMC8236118

[38] Sim G Kim Y Lee S Hahn J Kim J . Role of vitamin D in prevention of type 2 diabetes mellitus: A systematic review and meta−analysis. Exp Ther Med. (2024) 28:451. doi: 10.3892/etm.2024.12741, PMID: 39421597 PMC11484325

[39] Pordzik J Jakubik D Jarosz-Popek J Wicik Z Eyileten C De Rosa S . Significance of circulating microRNAs in diabetes mellitus type 2 and platelet reactivity: bioinformatic analysis and review. Cardiovasc Diabetol. (2019) 18:113. doi: 10.1186/s12933-019-0918-x, PMID: 31470851 PMC6716825

[40] Kautzky-Willer A Harreiter J Pacini G . Sex and gender differences in risk, pathophysiology and complications of type 2 diabetes mellitus. Endocr Rev. (2016) 37:278–316. doi: 10.1210/er.2015-1137, PMID: 27159875 PMC4890267

[41] Meerson A Najjar A Saad E Sbeit W Barhoum M Assy N . Sex differences in plasma microRNA biomarkers of early and complicated diabetes mellitus in Israeli Arab and jewish patients. Non-Coding RNA. (2019) 5:32. doi: 10.3390/ncrna5020032, PMID: 30959814 PMC6631160

[42] Kraczkowska W Stachowiak L Pławski A Jagodziński PP . Circulating miRNA as potential biomarkers for diabetes mellitus type 2: should we focus on searching for sex differences? J Appl Genet. (2022) 63:293–303. doi: 10.1007/s13353-021-00678-5, PMID: 34984663 PMC8979931

[43] Li Z Chyr J Jia Z Wang L Hu X Wu X . Identification of Hub Genes Associated with Hypertension and Their Interaction with miRNA Based on Weighted Gene Coexpression Network Analysis (WGCNA) Analysis. Med Sci Monit. (2020) 26. Available online at: https://www.medscimonit.com/abstract/index/idArt/923514 (Accessed October 20, 2025)., PMID: 32888289 10.12659/MSM.923514 PMC7491244

[44] Coenen-Stass AML Magen I Brooks T Ben-Dov IZ Greensmith L Hornstein E . Evaluation of methodologies for microRNA biomarker detection by next generation sequencing. RNA Biol. (2018) 15:1133–45. doi: 10.1080/15476286.2018.1514236, PMID: 30223713 PMC6161688

[45] Mestdagh P Hartmann N Baeriswyl L Andreasen D Bernard N Chen C . Evaluation of quantitative miRNA expression platforms in the microRNA quality control (miRQC) study. Nat Methods. (2014) 11:809–15. doi: 10.1038/nmeth.3014, PMID: 24973947

[46] Zampetaki A Kiechl S Drozdov I Willeit P Mayr U Prokopi M . Plasma microRNA profiling reveals loss of endothelial miR-126 and other microRNAs in type 2 diabetes. Circ Res. (2010) 107:810–7. doi: 10.1161/CIRCRESAHA.110.226357, PMID: 20651284

[47] Tonyan ZN Barbitoff YA Nasykhova YA Danilova MM Kozyulina PY Mikhailova AA . Plasma microRNA profiling in type 2 diabetes mellitus: A pilot study. Int J Mol Sci. (2023) 24:17406. doi: 10.3390/ijms242417406, PMID: 38139235 PMC10744218

[48] Kok MGM De Ronde MWJ Moerland PD Ruijter JM Creemers EE Pinto-Sietsma SJ . Small sample sizes in high-throughput miRNA screens: A common pitfall for the identification of miRNA biomarkers. Biomol Detect Quantif. (2018) 15:1–5. doi: 10.1016/j.bdq.2017.11.002, PMID: 29276692 PMC5737945

[49] Sunderland N Skroblin P Barwari T Huntley RP Lu R Joshi A . MicroRNA biomarkers and platelet reactivity: the clot thickens. Circ Res. (2017) 120:418–35. doi: 10.1161/CIRCRESAHA.116.309303, PMID: 28104774

