# Supplementary material for: Circulating microRNA signatures as potential biomarkers differentiating diabetic, prediabetic, and healthy individuals
Source: Front Endocrinol (Lausanne). 2025 Nov 4;16:1699100. doi: 10.3389/fendo.2025.1699100 (PMC12630337; doi:10.3389/fendo.2025.1699100)
Supplement: Supplementary file 1 [file Table1.docx]

Supplementary Materials:

**Table S1.** Circulating human microRNAs (hsa-miRs) with differential expression in pairwise group comparisons. (A) Diabetic vs. control groups; (B) pre-diabetic vs. control groups; and (C) diabetic vs. pre-diabetic groups. For (A) and (B), miRNAs with significantly different expression (FDR < 0.05) are listed. For (C), no miRNAs reached statistical significance after multiple testing correction (FDR < 0.05); therefore, the top 10 trend-level miRNAs with the lowest raw p-values are reported. Negative log₂FC values indicate downregulation in the first group listed.

Abbreviations: hsa-miR, human microRNA; log₂FC, log₂ fold change (expression ratio in diabetic vs. control, pre-diabetic vs. control, or diabetic vs. pre-diabetic groups); FDR, false discovery rate (adjusted for multiple testing).

A.

| **hsa-miR** | **log_2_ FC** | **FDR** |
| --- | --- | --- |
| hsa-miR-4708-5p | -0.62 | 9.37E-05 |
| hsa-miR-152-5p | -0.80 | 1.19E-03 |
| hsa-miR-1197 | -0.92 | 1.47E-03 |
| hsa-miR-4708-3p | -0.84 | 3.84E-03 |
| hsa-miR-125b-5p | -0.92 | 4.37E-03 |
| hsa-miR-6512-5p | -0.55 | 8.27E-03 |
| hsa-miR-4747-5p | -0.70 | 8.45E-03 |
| hsa-miR-20b-3p | 1.07 | 8.45E-03 |
| hsa-miR-135a-2-3p | -0.37 | 1.25E-02 |
| hsa-miR-6502-3p | -0.52 | 1.25E-02 |
| hsa-miR-708-3p | -0.83 | 1.25E-02 |
| hsa-miR-620 | -0.64 | 1.25E-02 |
| hsa-miR-6767-5p | -0.66 | 1.25E-02 |
| hsa-miR-5582-5p | -0.82 | 1.25E-02 |
| hsa-miR-4663 | 0.74 | 1.25E-02 |
| hsa-miR-2355-5p | -0.62 | 1.25E-02 |
| hsa-miR-6079 | 0.58 | 1.25E-02 |
| hsa-miR-4329 | -0.66 | 1.25E-02 |
| hsa-miR-4482-5p | -0.43 | 1.25E-02 |
| hsa-miR-516b-5p | 0.47 | 1.25E-02 |
| hsa-miR-362-3p | -0.63 | 1.25E-02 |
| hsa-miR-4704-3p | -0.72 | 1.25E-02 |
| hsa-miR-1185-1-3p | -0.76 | 1.25E-02 |
| hsa-miR-1296-5p | -0.62 | 1.25E-02 |
| hsa-miR-3918 | -0.93 | 1.25E-02 |
| hsa-miR-1284 | -0.99 | 1.33E-02 |
| hsa-miR-4524a-5p | -0.77 | 1.33E-02 |
| hsa-miR-4494 | -0.85 | 1.45E-02 |
| hsa-miR-545-5p | -1.08 | 1.46E-02 |
| hsa-miR-4524b-3p | -0.77 | 1.68E-02 |
| hsa-miR-3130-3p | -0.69 | 1.73E-02 |
| hsa-miR-6806-5p | -0.47 | 1.73E-02 |
| hsa-miR-3612 | -0.55 | 1.73E-02 |
| hsa-miR-600 | -0.65 | 1.73E-02 |
| hsa-miR-541-5p | 0.50 | 1.73E-02 |
| hsa-miR-6513-5p | 0.57 | 1.73E-02 |
| hsa-miR-593-3p | 1.27 | 1.77E-02 |
| hsa-miR-2277-5p | 0.51 | 1.77E-02 |
| hsa-miR-3130-5p | -0.68 | 1.77E-02 |
| hsa-miR-4796-5p | -0.91 | 1.79E-02 |
| hsa-miR-300 | -1.13 | 1.80E-02 |
| hsa-miR-1185-2-3p | -0.74 | 1.80E-02 |
| hsa-miR-8054 | -0.82 | 1.92E-02 |
| hsa-miR-4653-5p | 0.89 | 1.92E-02 |
| hsa-miR-3691-5p | -1.03 | 1.92E-02 |
| hsa-miR-641 | -0.84 | 1.92E-02 |
| hsa-miR-6872-3p | 1.11 | 1.92E-02 |
| hsa-miR-1273h-3p | -0.73 | 2.19E-02 |
| hsa-miR-5190 | -0.55 | 2.19E-02 |
| hsa-miR-10392-5p | 0.68 | 2.22E-02 |
| hsa-miR-628-5p | -1.10 | 2.34E-02 |
| hsa-miR-196b-3p | -0.93 | 2.34E-02 |
| hsa-miR-5197-3p | -1.08 | 2.34E-02 |
| hsa-miR-194-3p | 0.40 | 2.34E-02 |
| hsa-miR-4464 | -0.55 | 2.34E-02 |
| hsa-miR-1972 | -0.67 | 2.36E-02 |
| hsa-miR-4297 | -0.56 | 2.36E-02 |
| hsa-miR-489-3p | -0.67 | 2.36E-02 |
| hsa-miR-7973 | -0.73 | 2.55E-02 |
| hsa-miR-6730-3p | -0.86 | 2.76E-02 |
| hsa-miR-181a-3p | 0.75 | 2.77E-02 |
| hsa-miR-619-5p | 0.68 | 2.77E-02 |
| hsa-miR-6510-3p | -1.10 | 2.77E-02 |
| hsa-miR-1262 | -0.37 | 2.77E-02 |
| hsa-miR-541-3p | -0.55 | 2.77E-02 |
| hsa-miR-3714 | 0.60 | 2.77E-02 |
| hsa-miR-1843 | -0.95 | 2.77E-02 |
| hsa-miR-376b-5p | -0.64 | 2.77E-02 |
| hsa-miR-4261 | -0.65 | 2.77E-02 |
| hsa-miR-8065 | 0.64 | 2.77E-02 |
| hsa-miR-3149 | -0.48 | 2.77E-02 |
| hsa-miR-4451 | -0.84 | 2.97E-02 |
| hsa-miR-100-3p | -0.63 | 3.12E-02 |
| hsa-miR-4485-5p | 0.82 | 3.14E-02 |
| hsa-miR-3189-5p | -0.72 | 3.14E-02 |
| hsa-miR-651-5p | 0.85 | 3.14E-02 |
| hsa-miR-6726-5p | -0.49 | 3.14E-02 |
| hsa-let-7i-5p | 1.92 | 3.14E-02 |
| hsa-miR-6724-5p | 0.86 | 3.14E-02 |
| hsa-miR-346 | 0.38 | 3.14E-02 |
| hsa-miR-302d-5p | -0.51 | 3.14E-02 |
| hsa-miR-3937 | -0.86 | 3.14E-02 |
| hsa-miR-3170 | -0.50 | 3.14E-02 |
| hsa-miR-1273c | -1.03 | 3.14E-02 |
| hsa-miR-10399-5p | -0.42 | 3.14E-02 |
| hsa-miR-25-5p | -0.45 | 3.14E-02 |
| hsa-miR-6751-3p | -1.74 | 3.14E-02 |
| hsa-miR-212-5p | -0.51 | 3.14E-02 |
| hsa-miR-6780b-3p | -0.79 | 3.14E-02 |
| hsa-miR-3661 | 0.58 | 3.14E-02 |
| hsa-miR-4709-5p | -0.44 | 3.14E-02 |
| hsa-miR-4636 | 1.05 | 3.14E-02 |
| hsa-miR-626 | -0.63 | 3.14E-02 |
| hsa-miR-376c-5p | -0.63 | 3.21E-02 |
| hsa-miR-372-5p | -0.51 | 3.23E-02 |
| hsa-miR-1303 | -0.99 | 3.24E-02 |
| hsa-miR-7158-3p | -0.82 | 3.24E-02 |
| hsa-miR-5011-3p | 0.84 | 3.27E-02 |
| hsa-miR-93-5p | 1.56 | 3.27E-02 |
| hsa-miR-5694 | -0.51 | 3.28E-02 |
| hsa-miR-3134 | -0.92 | 3.28E-02 |
| hsa-miR-3660 | 0.56 | 3.28E-02 |
| hsa-miR-4711-5p | -0.50 | 3.38E-02 |
| hsa-miR-8485 | -0.75 | 3.45E-02 |
| hsa-miR-3908 | -0.31 | 3.70E-02 |
| hsa-miR-214-3p | -0.54 | 3.79E-02 |
| hsa-miR-2278 | 1.46 | 3.84E-02 |
| hsa-miR-4330 | -0.62 | 4.11E-02 |
| hsa-miR-6774-5p | -0.48 | 4.11E-02 |
| hsa-miR-194-5p | -0.57 | 4.11E-02 |
| hsa-miR-3622a-5p | 0.42 | 4.11E-02 |
| hsa-miR-221-3p | 1.70 | 4.12E-02 |
| hsa-miR-6784-3p | -0.72 | 4.15E-02 |
| hsa-miR-3120-5p | -0.43 | 4.16E-02 |
| hsa-miR-4736 | -0.45 | 4.26E-02 |
| hsa-miR-494-3p | 0.48 | 4.28E-02 |
| hsa-miR-4467 | 0.76 | 4.37E-02 |
| hsa-miR-3934-5p | 0.46 | 4.52E-02 |
| hsa-miR-12126 | -0.56 | 4.52E-02 |
| hsa-miR-134-5p | 0.58 | 4.65E-02 |
| hsa-miR-1324 | -0.42 | 4.65E-02 |
| hsa-miR-124-5p | 0.70 | 4.65E-02 |
| hsa-miR-224-5p | 0.58 | 4.65E-02 |
| hsa-miR-6820-5p | -0.90 | 4.72E-02 |
| hsa-miR-3115 | -0.74 | 4.72E-02 |
| hsa-miR-4477b | -0.54 | 4.72E-02 |
| hsa-miR-1266-5p | 1.22 | 4.87E-02 |
| hsa-miR-6131 | 0.90 | 4.87E-02 |
| hsa-miR-103a-1-5p | 1.21 | 4.92E-02 |
| hsa-miR-7844-5p | 0.99 | 4.94E-02 |
| hsa-miR-3664-3p | -0.95 | 4.94E-02 |

B.

| hsa-miR | log_2_FC | FDR |
| --- | --- | --- |
| hsa-miR-4429 | 0.68 | 1.74E-03 |
| hsa-miR-3661 | 1.12 | 1.74E-03 |
| hsa-miR-1295a | 1.02 | 1.74E-03 |
| hsa-miR-545-5p | -1.79 | 1.85E-03 |
| hsa-miR-1295b-5p | 0.95 | 3.06E-03 |
| hsa-miR-2116-3p | 1.33 | 3.06E-03 |
| hsa-miR-1277-5p | -1.88 | 3.06E-03 |
| hsa-miR-1197 | -1.14 | 3.20E-03 |
| hsa-miR-125b-5p | -1.19 | 5.69E-03 |
| hsa-miR-3132 | -1.05 | 6.83E-03 |
| hsa-miR-628-5p | -1.75 | 8.15E-03 |
| hsa-miR-3918 | -1.33 | 9.61E-03 |
| hsa-miR-1303 | -1.64 | 1.22E-02 |
| hsa-miR-3664-3p | -1.68 | 1.22E-02 |
| hsa-miR-135a-2-3p | -0.48 | 1.22E-02 |
| hsa-miR-545-3p | -1.25 | 1.22E-02 |
| hsa-miR-1273h-3p | -1.08 | 1.22E-02 |
| hsa-miR-3150b-3p | 0.74 | 1.22E-02 |
| hsa-miR-5582-5p | -1.09 | 1.31E-02 |
| hsa-miR-3129-5p | -1.37 | 1.31E-02 |
| hsa-miR-1910-5p | 0.79 | 1.36E-02 |
| hsa-miR-3937 | -1.36 | 1.36E-02 |
| hsa-miR-1284 | -1.33 | 1.46E-02 |
| hsa-miR-600 | -0.91 | 1.46E-02 |
| hsa-miR-4709-5p | -0.69 | 1.46E-02 |
| hsa-miR-627-5p | -1.06 | 1.46E-02 |
| hsa-miR-4708-3p | -0.94 | 1.46E-02 |
| hsa-miR-1277-3p | -1.30 | 1.46E-02 |
| hsa-miR-6820-5p | -1.49 | 1.46E-02 |
| hsa-miR-219a-2-3p | 0.64 | 1.52E-02 |
| hsa-miR-6779-3p | -1.10 | 1.58E-02 |
| hsa-miR-3939 | 0.84 | 1.59E-02 |
| hsa-miR-4708-5p | -0.56 | 1.59E-02 |
| hsa-miR-449c-5p | -1.06 | 1.59E-02 |
| hsa-miR-193b-3p | 0.70 | 1.59E-02 |
| hsa-miR-3975 | 0.65 | 1.60E-02 |
| hsa-miR-5590-3p | -1.09 | 1.65E-02 |
| hsa-miR-6831-3p | -0.55 | 1.65E-02 |
| hsa-miR-568 | -1.31 | 1.68E-02 |
| hsa-miR-4635 | 0.93 | 1.82E-02 |
| hsa-miR-6769a-3p | -1.05 | 1.84E-02 |
| hsa-miR-7155-3p | 0.79 | 1.84E-02 |
| hsa-miR-1304-3p | 1.39 | 1.87E-02 |
| hsa-miR-1236-3p | -0.98 | 1.96E-02 |
| hsa-miR-8485 | -1.11 | 2.19E-02 |
| hsa-miR-3183 | 0.81 | 2.27E-02 |
| hsa-miR-3934-5p | 0.70 | 2.28E-02 |
| hsa-miR-5002-3p | 1.03 | 2.28E-02 |
| hsa-miR-548ac | 0.84 | 2.28E-02 |
| hsa-miR-6751-3p | -2.50 | 2.28E-02 |
| hsa-miR-3150a-5p | 0.64 | 2.28E-02 |
| hsa-miR-4776-5p | 0.62 | 2.28E-02 |
| hsa-miR-5197-3p | -1.45 | 2.30E-02 |
| hsa-miR-2277-5p | 0.64 | 2.30E-02 |
| hsa-miR-620 | -0.75 | 2.30E-02 |
| hsa-miR-1224-3p | -0.97 | 2.30E-02 |
| hsa-miR-1185-1-3p | -0.91 | 2.30E-02 |
| hsa-miR-4524b-3p | -0.96 | 2.30E-02 |
| hsa-miR-548i | 0.75 | 2.30E-02 |
| hsa-miR-4683 | 0.71 | 2.30E-02 |
| hsa-miR-519c-3p | -0.98 | 2.30E-02 |
| hsa-miR-1468-3p | -1.49 | 2.30E-02 |
| hsa-miR-6784-3p | -1.06 | 2.30E-02 |
| hsa-miR-4524a-5p | -0.93 | 2.30E-02 |
| hsa-miR-4451 | -1.16 | 2.30E-02 |
| hsa-miR-6780b-3p | -1.11 | 2.30E-02 |
| hsa-miR-1185-2-3p | -0.94 | 2.30E-02 |
| hsa-miR-544a | -1.24 | 2.30E-02 |
| hsa-miR-212-5p | -0.71 | 2.45E-02 |
| hsa-miR-6767-5p | -0.76 | 2.45E-02 |
| hsa-miR-300 | -1.42 | 2.45E-02 |
| hsa-miR-6794-3p | -1.03 | 2.45E-02 |
| hsa-miR-124-5p | 1.02 | 2.45E-02 |
| hsa-miR-6726-5p | -0.68 | 2.45E-02 |
| hsa-miR-6512-5p | -0.57 | 2.50E-02 |
| hsa-miR-4482-5p | -0.50 | 2.55E-02 |
| hsa-miR-489-3p | -0.87 | 2.56E-02 |
| hsa-miR-6833-3p | -0.78 | 2.56E-02 |
| hsa-miR-215-3p | -0.89 | 2.56E-02 |
| hsa-miR-621 | 0.56 | 2.56E-02 |
| hsa-miR-6510-3p | -1.46 | 2.57E-02 |
| hsa-miR-3189-5p | -0.97 | 2.60E-02 |
| hsa-miR-6730-3p | -1.12 | 2.66E-02 |
| hsa-miR-331-5p | 1.07 | 2.68E-02 |
| hsa-miR-3691-5p | -1.27 | 2.97E-02 |
| hsa-miR-541-5p | 0.59 | 2.97E-02 |
| hsa-miR-1843 | -1.24 | 2.98E-02 |
| hsa-miR-653-5p | -0.94 | 2.99E-02 |
| hsa-miR-6873-3p | -1.69 | 2.99E-02 |
| hsa-miR-4753-3p | -1.26 | 2.99E-02 |
| hsa-miR-548ah-5p | 1.08 | 2.99E-02 |
| hsa-miR-1322 | 0.55 | 2.99E-02 |
| hsa-miR-182-3p | 0.72 | 2.99E-02 |
| hsa-miR-3193 | 0.75 | 3.02E-02 |
| hsa-miR-6778-3p | 0.96 | 3.03E-02 |
| hsa-miR-100-3p | -0.82 | 3.05E-02 |
| hsa-miR-513b-3p | -1.03 | 3.06E-02 |
| hsa-miR-376a-5p | -0.84 | 3.06E-02 |
| hsa-miR-3611 | -1.18 | 3.06E-02 |
| hsa-miR-6736-3p | -0.53 | 3.06E-02 |
| hsa-miR-1179 | -0.85 | 3.06E-02 |
| hsa-miR-4503 | -0.81 | 3.06E-02 |
| hsa-miR-7158-3p | -1.10 | 3.06E-02 |
| hsa-miR-4477b | -0.77 | 3.08E-02 |
| hsa-miR-6746-3p | -0.84 | 3.08E-02 |
| hsa-miR-6768-3p | 0.53 | 3.10E-02 |
| hsa-miR-497-3p | -0.65 | 3.16E-02 |
| hsa-miR-32-3p | -0.61 | 3.18E-02 |
| hsa-miR-4329 | -0.73 | 3.25E-02 |
| hsa-miR-12133 | -0.88 | 3.25E-02 |
| hsa-miR-3622a-5p | 0.56 | 3.39E-02 |
| hsa-miR-1324 | -0.58 | 3.43E-02 |
| hsa-miR-4790-3p | 0.61 | 3.44E-02 |
| hsa-miR-297 | -0.89 | 3.44E-02 |
| hsa-miR-7973 | -0.90 | 3.48E-02 |
| hsa-miR-3146 | -1.13 | 3.63E-02 |
| hsa-miR-6878-3p | -1.11 | 3.69E-02 |
| hsa-miR-4703-5p | -0.95 | 3.81E-02 |
| hsa-miR-6835-3p | -0.85 | 4.00E-02 |
| hsa-miR-1273c | -1.32 | 4.02E-02 |
| hsa-miR-1204 | -0.66 | 4.14E-02 |
| hsa-miR-4746-3p | 0.75 | 4.14E-02 |
| hsa-miR-1207-3p | 0.81 | 4.15E-02 |
| hsa-miR-6879-3p | 0.54 | 4.31E-02 |
| hsa-miR-6827-3p | -1.05 | 4.34E-02 |
| hsa-let-7f-2-3p | -0.75 | 4.37E-02 |
| hsa-miR-4529-3p | 0.73 | 4.38E-02 |
| hsa-miR-219b-5p | 0.55 | 4.38E-02 |
| hsa-miR-1293 | 1.01 | 4.38E-02 |
| hsa-miR-520e-3p | -1.41 | 4.38E-02 |
| hsa-miR-4789-5p | -0.55 | 4.47E-02 |
| hsa-miR-4297 | -0.65 | 4.69E-02 |
| hsa-miR-8080 | 0.83 | 4.69E-02 |
| hsa-miR-4434 | -0.79 | 4.71E-02 |
| hsa-miR-4326 | -0.67 | 4.71E-02 |
| hsa-miR-141-3p | -0.77 | 4.75E-02 |
| hsa-miR-194-3p | 0.46 | 4.79E-02 |
| hsa-miR-3622b-3p | 0.59 | 4.79E-02 |
| hsa-miR-2467-5p | -0.71 | 4.93E-02 |
| hsa-miR-135b-5p | -1.13 | 4.95E-02 |
| hsa-miR-302d-5p | -0.62 | 4.95E-02 |

**C.**

| hsa-miR | log_2_FC | FDR |
| --- | --- | --- |
| hsa-miR-4776-5p | -0.65 | 0.22 |
| hsa-miR-6778-3p | -1.02 | 0.30 |
| hsa-miR-5002-3p | -0.99 | 0.32 |
| hsa-miR-1304-3p | -1.23 | 0.33 |
| hsa-miR-580-3p | -0.88 | 0.33 |
| hsa-miR-548a-3p | -0.48 | 0.33 |
| hsa-miR-4790-3p | -0.58 | 0.33 |
| hsa-miR-4724-3p | -0.57 | 0.33 |
| hsa-miR-193b-3p | -0.57 | 0.33 |
| hsa-miR-1207-3p | -0.78 | 0.33 |

**Table S2.** Differentially expressed human microRNAs (hsa-miRs) in (A) diabetic vs. control and (B) pre-diabetic vs. control groups, compared with the candidate list from Zhu et al. (2023). A total of 16 hsa-miRs identified as potential biomarkers for type 2 diabetes are included. Abbreviations: hsa-miR, human microRNA; log₂FC, log₂ fold change (expression ratio in diabetic or pre-diabetic vs. control groups); FDR, false discovery rate (adjusted for multiple testing). *Order of priority based on significance and relevance. **FDR-adjusted p-value: q<0.05.

A.

| Zhu et al. 2023 | Matching has-miRs | |
| --- | --- | --- |
| Top 16 has-miRs* | log_2_FC | FDR |
| hsa-miR-29a-3p | -0.37 | 0.56 |
| hsa-miR-221-3p | 1.70 | 0.04** |
| hsa-miR-126-3p | 0.10 | 0.93 |
| hsa-miR-26a-5p | 0.09 | 0.94 |
| hsa-miR-503-5p | 0.26 | 0.52 |
| hsa-miR-100-5p | -0.07 | 0.89 |
| hsa-miR-101-3p | 0.07 | 0.90 |
| hsa-miR-103a-3p | 0.62 | 0.45 |
| hsa-miR-122-5p | NA | NA |
| hsa-miR-199a-3p | 0.69 | 0.48 |
| hsa-miR-30b-5p | 0.11 | 0.82 |
| hsa-miR-130a-3p | 0.25 | 0.66 |
| hsa-miR-143-3p | -0.04 | 0.94 |
| hsa-miR-145-5p | -0.02 | 0.96 |
| hsa-miR-19a-3p | -0.02 | 0.99 |
| hsa-miR-311-3p | NA | NA |

B.

| Zhu et al. 2023 | Matching has-miRs | |
| --- | --- | --- |
| Top 16 has-miRs* | **log_2_FC** | **FDR** |
| hsa-miR-29a-3p | -0.62 | 0.43 |
| hsa-miR-221-3p | 0.25 | 0.88 |
| hsa-miR-126-3p | -0.70 | 0.46 |
| hsa-miR-26a-5p | -0.47 | 0.70 |
| hsa-miR-503-5p | 0.70 | 0.12 |
| hsa-miR-100-5p | 0.35 | 0.46 |
| hsa-miR-101-3p | -0.30 | 0.57 |
| hsa-miR-103a-3p | -0.89 | 0.39 |
| hsa-miR-122-5p | NA | NA |
| hsa-miR-199a-3p | -0.57 | 0.67 |
| hsa-miR-30b-5p | 0.01 | 0.99 |
| hsa-miR-130a-3p | -0.41 | 0.56 |
| hsa-miR-143-3p | 0.03 | 0.96 |
| hsa-miR-145-5p | -0.15 | 0.67 |
| hsa-miR-19a-3p | -0.59 | 0.50 |
| hsa-miR-311-3p | NA | NA |

**Table S3.** Differentially expressed human microRNAs (hsa-miRs) across sex and glycaemic state. Differential expression of circulating hsa-miRs was assessed across multiple group comparisons, adjusted for sex where indicated. Comparisons include: (1) males vs. females regardless of disease status; (2) diabetic males vs. females; (3) diabetic vs. control individuals accounting for sex; (4) diabetic vs. pre-diabetic individuals accounting for sex; and (5) pre-diabetic vs. control individuals accounting for sex. The table lists hsa-miR identifiers, log₂ fold change (log₂FC), and false discovery rate (FDR). Positive log₂FC values indicate upregulation in the first group. Statistically significant differential expression (FDR < 0.05) highlights sex-specific and glycaemic state–dependent patterns. Abbreviations: hsa-miR, human microRNA; log₂FC, log₂ fold change; FDR, false discovery rate.

|  | Comparison | hsa-miR | log_2_FC | FDR |
| --- | --- | --- | --- | --- |
| 1 | Males vs. Females, regardless of disease status | hsa-miR-4289 | 2.41 | 9.78E-05 |
|  |  | hsa-miR-4432 | 2.59 | 0.002 |
|  |  | hsa-miR-514a-5p | 1.70 | 0.004 |
| 2 | Diabetic Males vs. Females | hsa-miR-4289 | -2.50 | 0.011 |
|  |  | hsa-miR-4432 | -2.94 | 0.011 |
| 3 | Diabetic vs. Control, accounting for sex | hsa-miR-6131 | 0.002 | 1.491 |
|  |  | hsa-miR-4708-5p | 0.002 | -0.732 |
|  |  | hsa-miR-221-3p | 0.004 | 2.469 |
|  |  | hsa-miR-20b-3p | 0.005 | 1.366 |
|  |  | hsa-miR-4652-3p | 0.011 | 1.580 |
|  |  | hsa-miR-6806-5p | 0.016 | -0.687 |
|  |  | hsa-miR-7844-5p | 0.016 | 1.441 |
|  |  | hsa-miR-93-5p | 0.018 | 2.184 |
|  |  | hsa-miR-4653-5p | 0.021 | 1.206 |
|  |  | hsa-miR-12130 | 0.022 | 1.534 |
|  |  | hsa-miR-6751-3p | 0.025 | -2.472 |
|  |  | hsa-let-7d-5p | 0.025 | 2.211 |
|  |  | hsa-miR-17-5p | 0.027 | 1.939 |
|  |  | hsa-miR-651-5p | 0.027 | 1.210 |
|  |  | hsa-miR-509-5p | 0.029 | 1.070 |
|  |  | hsa-miR-5190 | 0.030 | -0.756 |
|  |  | hsa-let-7i-5p | 0.031 | 2.522 |
|  |  | hsa-miR-744-5p | 0.031 | 1.756 |
|  |  | hsa-miR-593-3p | 0.031 | 1.595 |
|  |  | hsa-miR-4708-3p | 0.032 | -0.940 |
|  |  | hsa-miR-1296-5p | 0.039 | -0.749 |
|  |  | hsa-miR-494-5p | 0.045 | 0.930 |
|  |  | hsa-miR-541-5p | 0.045 | 0.636 |
|  |  | hsa-miR-4485-5p | 0.045 | 1.071 |
|  |  | hsa-miR-4747-5p | 0.045 | -0.797 |
|  |  | hsa-miR-199a-5p | 0.045 | 1.061 |
|  |  | hsa-miR-520e-3p | 0.046 | -1.406 |
|  |  | hsa-miR-125b-5p | 0.046 | -0.969 |
|  |  | hsa-miR-181a-3p | 0.047 | 0.993 |
|  |  | hsa-miR-4677-5p | 0.047 | 0.960 |
|  |  | hsa-miR-6767-5p | 0.047 | -0.769 |
|  |  | hsa-miR-6813-5p | 0.047 | 0.948 |
|  |  | hsa-miR-5694 | 0.047 | -0.726 |
|  |  | hsa-miR-5087 | 0.047 | 0.561 |
|  |  | hsa-miR-10392-5p | 0.047 | 0.858 |
|  |  | hsa-miR-6780b-3p | 0.047 | -0.998 |
|  |  | hsa-miR-8076 | 0.047 | -1.306 |
|  |  | hsa-miR-196b-3p | 0.047 | -1.171 |
|  |  | hsa-miR-4667-3p | 0.047 | -0.886 |
|  |  | hsa-miR-126-3p | 0.049 | 2.181 |
| 4 | Diabetic vs. Prediabetic, accounting for sex | hsa-miR-4800-5p | -3.13 | 0.002 |
|  |  | hsa-miR-5002, -3p | -1.63 | 0.015 |
| 5 | Prediabetic vs. Control, accounting for sex | hsa-miR-4429 | 0.016 | 0.837 |
|  |  | hsa-miR-4800-5p | 0.016 | 2.675 |
|  |  | hsa-miR-4693-3p | 0.024 | 2.869 |
|  |  | hsa-miR-5002-3p | 0.024 | 1.496 |
|  |  | hsa-miR-3661 | 0.044 | 1.273 |
|  |  | hsa-miR-1277-5p | 0.044 | -2.219 |
|  |  | hsa-miR-1204 | 0.044 | -1.037 |

**Table S4.** Summary of significantly differentially expressed human microRNAs (hsa-miRs; FDR < 0.05) in diabetic vs. control and aspirin-use comparisons.
Differential expression of circulating hsa-miRs was assessed in three comparisons: (1) diabetic patients taking aspirin vs. healthy controls; (2) diabetic patients not taking aspirin vs. healthy controls; and (3) diabetic patients taking aspirin vs. diabetic patients not taking aspirin. The table lists hsa-miR identifiers, log₂ fold change (log₂FC), and false discovery rate (FDR). Positive log₂FC values indicate upregulation in the first group. No hsa-miRs reached statistical significance in comparison (3). Abbreviations: hsa-miR, human microRNA; log₂FC, log₂ fold change; FDR, false discovery rate.

| Comparison | hsa-miR | log_2_FC | FDR |
| --- | --- | --- | --- |
| 1. Diabetic (Aspirin) vs Control | hsa-miR-4708-5p | -0.609 | 6.240E-05 |
|  | hsa-miR-6513-5p | 0.753 | 0.016 |
|  | hsa-miR-20b-3p | 1.223 | 0.020 |
|  | hsa-miR-8054 | -0.951 | 0.020 |
|  | hsa-miR-3149 | -0.697 | 0.020 |
|  | hsa-miR-1197 | -0.934 | 0.020 |
|  | hsa-miR-619-5p | 0.883 | 0.020 |
|  | hsa-miR-4663 | 0.871 | 0.023 |
|  | hsa-miR-620 | -0.675 | 0.023 |
|  | hsa-miR-450a-1-3p | -0.789 | 0.023 |
|  | hsa-miR-6512-5p | -0.611 | 0.024 |
|  | hsa-miR-125b-5p | -0.947 | 0.025 |
|  | hsa-miR-642a-5p | -0.542 | 0.025 |
|  | hsa-miR-3937 | -1.163 | 0.025 |
|  | hsa-miR-1262 | -0.479 | 0.025 |
|  | hsa-miR-106b-3p | 0.882 | 0.025 |
|  | hsa-miR-6872-3p | 1.359 | 0.025 |
|  | hsa-miR-6073 | 3.188 | 0.025 |
|  | hsa-miR-3189-5p | -0.951 | 0.025 |
|  | hsa-miR-516b-5p | 0.537 | 0.025 |
|  | hsa-miR-4690-3p | 0.595 | 0.025 |
|  | hsa-miR-6767-5p | -0.706 | 0.025 |
|  | hsa-miR-1296-5p | -0.705 | 0.026 |
|  | hsa-miR-3612 | -0.656 | 0.026 |
|  | hsa-miR-6079 | 0.614 | 0.026 |
|  | hsa-miR-4451 | -1.081 | 0.029 |
|  | hsa-miR-3661 | 0.621 | 0.029 |
|  | hsa-miR-6753-3p | -0.536 | 0.029 |
|  | hsa-miR-3130-3p | -0.832 | 0.029 |
|  | hsa-miR-7158-3p | -1.051 | 0.029 |
|  | hsa-miR-6820-5p | -1.228 | 0.029 |
|  | hsa-miR-3130-5p | -0.832 | 0.029 |
|  | hsa-miR-8065 | 0.750 | 0.029 |
|  | hsa-miR-3918 | -0.988 | 0.032 |
|  | hsa-miR-628-5p | -1.286 | 0.032 |
|  | hsa-miR-134-5p | 0.790 | 0.032 |
|  | hsa-miR-6806-5p | -0.527 | 0.032 |
|  | hsa-miR-4747-5p | -0.730 | 0.032 |
|  | hsa-miR-4653-5p | 1.049 | 0.036 |
|  | hsa-miR-3912-3p | -0.769 | 0.040 |
|  | hsa-miR-135a-2-3p | -0.404 | 0.040 |
|  | hsa-miR-1245a | 1.077 | 0.040 |
|  | hsa-miR-548c-3p | -0.499 | 0.043 |
|  | hsa-miR-7848-3p | 1.172 | 0.043 |
|  | hsa-miR-375-3p | 0.977 | 0.043 |
|  | hsa-miR-4467 | 0.988 | 0.045 |
|  | hsa-miR-194-3p | 0.401 | 0.045 |
|  | hsa-miR-3173-5p | 1.215 | 0.045 |
|  | hsa-miR-494-3p | 0.630 | 0.045 |
|  | hsa-miR-1273c | -1.014 | 0.045 |
|  | hsa-miR-4799-3p | 1.107 | 0.045 |
|  | hsa-miR-3660 | 0.715 | 0.045 |
|  | hsa-miR-4708-3p | -0.690 | 0.045 |
|  | hsa-miR-545-5p | -1.168 | 0.045 |
|  | hsa-miR-2355-5p | -0.643 | 0.045 |
|  | hsa-miR-4263 | 0.910 | 0.045 |
|  | hsa-miR-4704-3p | -0.751 | 0.045 |
|  | hsa-miR-5011-3p | 1.035 | 0.045 |
|  | hsa-miR-8485 | -0.908 | 0.045 |
|  | hsa-miR-10392-5p | 0.702 | 0.045 |
|  | hsa-miR-181b-3p | 1.064 | 0.045 |
|  | hsa-miR-7156-3p | 0.655 | 0.045 |
|  | hsa-miR-433-5p | 1.006 | 0.045 |
|  | hsa-miR-106b-5p | 0.800 | 0.048 |
|  | hsa-miR-3664-3p | -1.218 | 0.048 |
|  | hsa-miR-4738-3p | 1.065 | 0.048 |
|  | hsa-miR-5582-5p | -0.823 | 0.048 |
|  | hsa-miR-1185-1-3p | -0.717 | 0.048 |
| (2) Diabetic (No Aspirin) vs Control | hsa-miR-4708-5p | –0.541 | 2.27E-04 |
|  | hsa-miR-4482-5p | –0.532 | 0.033 |
|  | hsa-miR-152-5p | –0.769 | 0.033 |
| (3) Diabetic (Aspirin) vs Diabetic (No Aspirin) | **None significant** | | |

**Table S5.** Significant Pearson correlations among clinical and laboratory variables by participant subgroup. The table summarizes the significant Pearson correlation coefficients (r, p < 0.05) between clinical and laboratory variables, organized by subgroup: all participants, controls, diabetics, females, and males. The analysis highlights associations in glucose metabolism, lipid profile, platelet function, renal markers, and vitamin D status. Each entry lists the variable pair, correlation strength, and statistical significance. Abbreviations: PLT_X10³/μL, platelet count (×10³/μL); PRP_PLT_X10³, platelet-rich plasma platelet count (×10³/μL); HbA1c, glycated hemoglobin; glucose, blood glucose; Diabetes_duration, years since diagnosis; vitD25_nmol_L, 25-hydroxyvitamin D (nmol/L); ADP1_agg_slope, platelet aggregation slope in response to adenosine diphosphate (ADP, 1 µM); HDL, high-density lipoprotein cholesterol; trig, triglycerides; cholesterol, total cholesterol; collagen_agg_max, maximum aggregation in response to collagen; creatinine, serum creatinine; MPV_fL, mean platelet volume (femtoliters); Ca, serum calcium; BMI, body mass index; age, participant age. Column headers: subset, participant subgroup; var1, first variable; var2, second variable; cor, Pearson correlation coefficient (r); p, p-value.

| subset | var1 | var2 | cor | p |
| --- | --- | --- | --- | --- |
| All | PLT_X10_3_ul | PRP_PLT_X10_3 | 0.86 | 5.88E-08 |
| All | Diabetes_duration | HbA1c | 0.82 | 1.41E-06 |
| All | HbA1c | glucose | 0.78 | 2.03E-05 |
| All | Diabetes_duration | glucose | 0.76 | 4.74E-05 |
| All | trig | HDL | -0.56 | 0.006 |
| All | vitD25_nmol_L | glucose | -0.52 | 0.013 |
| All | cholesterol | HDL | 0.5 | 0.017 |
| All | age | cholesterol | -0.5 | 0.018 |
| All | glucose | HDL | -0.49 | 0.020 |
| All | vitD25_nmol_L | ADP1_agg_slope | -0.49 | 0.021 |
| All | cholesterol | collagen_agg_max | -0.48 | 0.027 |
| All | glucose | ADP1_agg_slope | 0.46 | 0.032 |
| All | HbA1c | vitD25_nmol_L | -0.45 | 0.034 |
| All | HbA1c | HDL | -0.45 | 0.036 |
| Control | PLT_X10_3_ul | PRP_PLT_X10_3 | 0.85 | 0.008 |
| Control | trig | createnin | 0.87 | 0.011 |
| Control | glucose | PLT_X10_3_ul | -0.87 | 0.011 |
| Control | age | PRP_PLT_X10_3 | -0.76 | 0.030 |
| Control | HbA1c | Ca | 0.76 | 0.046 |
| Diabetic | PLT_X10_3_ul | PRP_PLT_X10_3 | 0.88 | 0.000 |
| Diabetic | createnin | MPV_fL | -0.72 | 0.004 |
| Diabetic | glucose | ADP1_agg_slope | 0.7 | 0.005 |
| Diabetic | HbA1c | collagen_agg_max | 0.68 | 0.008 |
| Diabetic | Diabetes_duration | createnin | 0.61 | 0.020 |
| Diabetic | trig | HDL | -0.57 | 0.033 |
| Diabetic | age | cholesterol | -0.55 | 0.041 |
| Diabetic | Diabetes_duration | MPV_fL | -0.54 | 0.045 |
| Female | PLT_X10_3_ul | PRP_PLT_X10_3 | 0.82 | 0.001 |
| Female | HbA1c | glucose | 0.86 | 0.001 |
| Female | Diabetes_duration | HbA1c | 0.82 | 0.001 |
| Female | Diabetes_duration | glucose | 0.8 | 0.003 |
| Female | vitD25_nmol_L | createnin | 0.64 | 0.034 |
| Female | Ca | MPV_fL | -0.62 | 0.040 |
| Female | vitD25_nmol_L | Ca | 0.61 | 0.045 |
| Male | PLT_X10_3_ul | PRP_PLT_X10_3 | 0.89 | 2.23E-04 |
| Male | Diabetes_duration | HbA1c | 0.81 | 0.003 |
| Male | vitD25_nmol_L | ADP1_agg_slope | -0.75 | 0.008 |
| Male | HbA1c | glucose | 0.68 | 0.021 |
| Male | Diabetes_duration | glucose | 0.68 | 0.022 |
| Male | trig | HDL | -0.67 | 0.024 |
| Male | age | Ca | -0.63 | 0.036 |
| Male | age | trig | -0.62 | 0.041 |
| Male | BMI | MPV_fL | 0.62 | 0.044 |
| Male | Ca | MPV_fL | 0.61 | 0.048 |
